# The putative proton-coupled organic cation antiporter is involved in uptake of triptans into human brain capillary endothelial cells

**DOI:** 10.1186/s12987-024-00544-6

**Published:** 2024-05-06

**Authors:** Nana Svane, Alberte Bay Villekjær Pedersen, Anne Rodenberg, Burak Ozgür, Lasse Saaby, Christoffer Bundgaard, Mie Kristensen, Peer Tfelt-Hansen, Birger Brodin

**Affiliations:** 1 Department of Pharmacy, University of Copenhagen, Copenhagen, Denmark; 2 Biotherapeutic Discovery, H. Lundbeck A/S, Valby, Denmark; 3 Bioneer: FARMA, Bioneer A/S, Copenhagen, Denmark; 4 Translational DMPK, H. Lundbeck A/S, Valby, Denmark; 5 Danish Headache Center, Department of Neurology, Rigshospitalet-Glostrup, University of Copenhagen, Glostrup, Denmark

**Keywords:** Blood–brain barrier, Capillary endothelium, Triptans, Migraine treatment, Proton-coupled organic cation antiporter, P-glycoprotein, Oxycodone transporter, Active uptake

## Abstract

**Background:**

Triptans are anti-migraine drugs with a potential central site of action. However, it is not known to what extent triptans cross the blood–brain barrier (BBB). The aim of this study was therefore to determine if triptans pass the brain capillary endothelium and investigate the possible underlying mechanisms with focus on the involvement of the putative proton-coupled organic cation (H^+^/OC) antiporter. Additionally, we evaluated whether triptans interacted with the efflux transporter, P-glycoprotein (P-gp).

**Methods:**

We investigated the cellular uptake characteristics of the prototypical H^+^/OC antiporter substrates, pyrilamine and oxycodone, and seven different triptans in the human brain microvascular endothelial cell line, hCMEC/D3. Triptan interactions with P-gp were studied using the IPEC-J2 MDR1 cell line. Lastly, in vivo neuropharmacokinetic assessment of the unbound brain-to-plasma disposition of eletriptan was conducted in wild type and mdr1a/1b knockout mice.

**Results:**

We demonstrated that most triptans were able to inhibit uptake of the H^+^/OC antiporter substrate, pyrilamine, with eletriptan emerging as the strongest inhibitor. Eletriptan, almotriptan, and sumatriptan exhibited a pH-dependent uptake into hCMEC/D3 cells. Eletriptan demonstrated saturable uptake kinetics with an apparent K_m_ of 89 ± 38 µM and a J_max_ of 2.2 ± 0.7 nmol·min^−1^·mg protein^−1^ (*n* = 3). Bidirectional transport experiments across IPEC-J2 MDR1 monolayers showed that eletriptan is transported by P-gp, thus indicating that eletriptan is both a substrate of the H^+^/OC antiporter and P-gp. This was further confirmed in vivo, where the unbound brain-to-unbound plasma concentration ratio (K_p,uu_) was 0.04 in wild type mice while the ratio rose to 1.32 in mdr1a/1b knockout mice.

**Conclusions:**

We have demonstrated that the triptan family of compounds possesses affinity for the H^+^/OC antiporter proposing that the putative H^+^/OC antiporter plays a role in the BBB transport of triptans, particularly eletriptan. Our i*n vivo* studies indicate that eletriptan is subjected to simultaneous brain uptake and efflux, possibly facilitated by the putative H^+^/OC antiporter and P-gp, respectively. Our findings offer novel insights into the potential central site of action involved in migraine treatment with triptans and highlight the significance of potential transporter related drug-drug interactions.

**Graphical Abstract:**

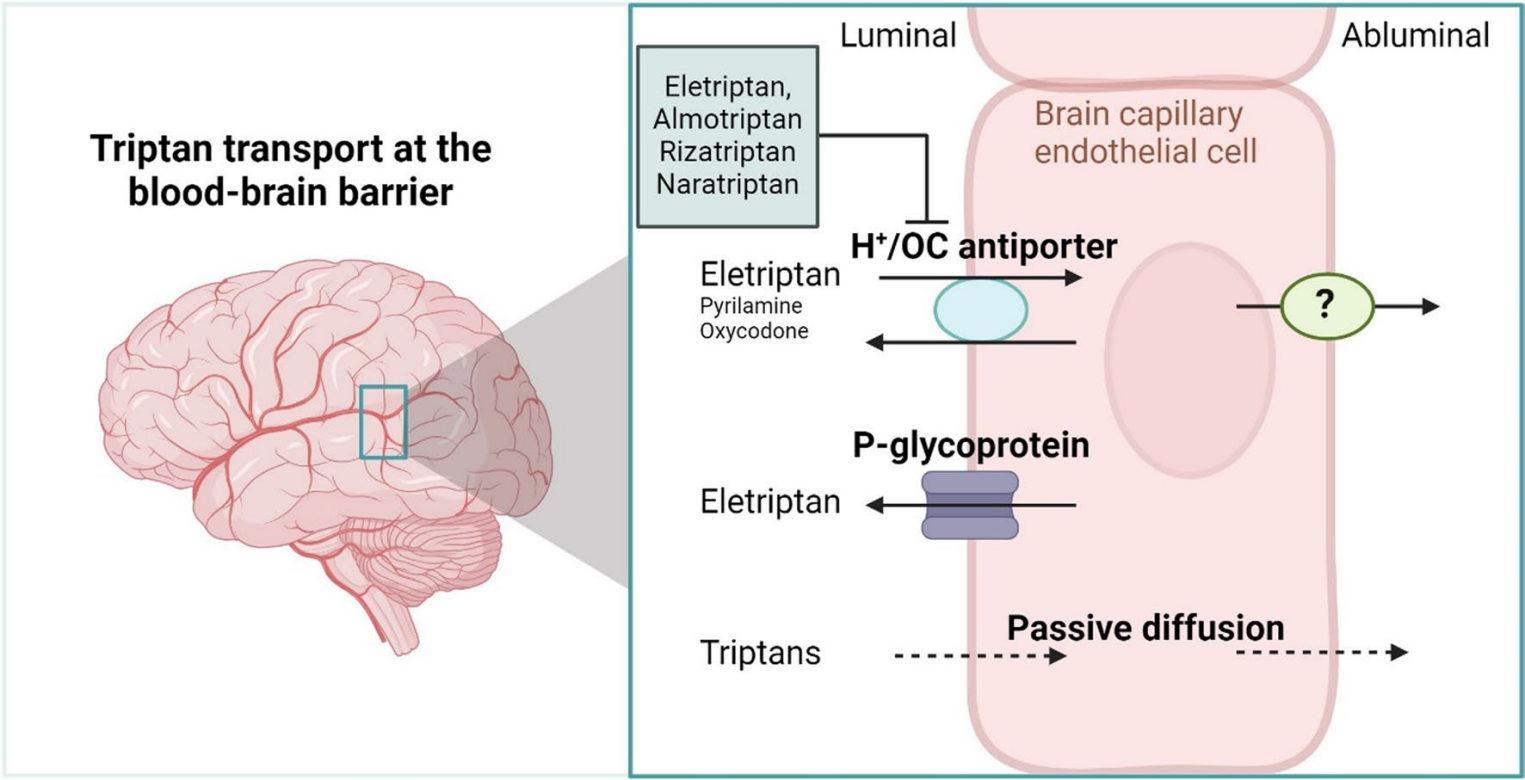

**Supplementary Information:**

The online version contains supplementary material available at 10.1186/s12987-024-00544-6.

## Background

Migraine is a common chronic neurological disorder affecting more than 10% of the population [1, 2]. Triptans are a class of tryptamine-based compounds used in the treatment of migraine. The seven triptans in clinical use are sumatriptan, almotriptan, eletriptan, frovatriptan, naratriptan, rizatriptan, and zolmitriptan [3]. Triptans are potent agonists of the 5HT_1B/1D_ receptors that are found on smooth muscle cells in the intracranial extracerebral vasculature, where activation results in the constriction of the vasculature and hereby relief of migraine symptoms [4, 5]. Furthermore, triptans inhibit the release of vasodilating and inflammatory substances from the trigeminal nerve [4, 5]. In addition to these peripheral effects, several factors indicate that triptans may act on 5HT_1B/1D_ receptors within the central nervous system (CNS), but the mechanism by which this occurs is poorly understood [6, 7].

Triptans are relatively hydrophilic compounds carrying a positive charge at physiologically relevant pH. As a consequence, triptans are expected to exhibit a limited ability to cross the restrictive blood–brain barrier (BBB) by passive diffusion [7]. However, common central side effects of triptans such as dizziness, fatigue, somnolence, and confusion, suggest that triptans enter the brain parenchyma to some extent [8, 9]. In addition, several preclinical studies have demonstrated a general disposition of triptans in the brain, as well as central 5HT_1B/1D_ receptor activation after systemic administration [10–16]. Both of these observations indicate that triptans cross the BBB, possibly facilitated by carrier-mediated transport when considering the hydrophilic nature of this class of drug compounds.

A putative proton-coupled organic cation (H^+^/OC) antiporter was discovered in the 1980’s based on its function [17, 18]. More recent studies have provided functional and molecular identification of the H^+^/OC antiporter, which differentiate from other known transporters [19–22]. The H^+^/OC antiporter is suggested to transport substrates such as histamine receptor antagonists (pyrilamine and diphenhydramine) [19, 21, 23, 24], opioid agonists (oxycodone and tramadol) [19, 25], memantine (an NMDA receptor antagonist) [26], pramipexol (a dopamine 2 receptor agonist) [27], and nicotinic acetylcholine receptor agonists (nicotine and varenicline) [28, 29]. A number of these substrates are found to not only cross the BBB, but also to accumulate in the brain, as indicated by an unbound brain-to-unbound plasma partitioning coefficient (K_p,uu_) above 1 [29–32]. A common trait among these H^+^/OC antiporter substrates, is that they are small molecules bearing a secondary or tertiary amine moiety, which is positively charged at physiological relevant pH [33, 34].

All triptans possess a secondary or tertiary amine moiety, which is positively charged at physiological pH, a common trait of H^+^/OC antiporter substrates. We therefore hypothesized that triptans could be substrates of the H^+^/OC antiporter, which could have significant implications for their ability to cross the BBB. The aim of the present study was to investigate the potential involvement of the H^+^/OC antiporter in the uptake of triptans into brain capillary endothelial cells. Uptake and transport of triptans was investigated in the human brain microvascular endothelial cell line hCMEC/D3, which previously have been shown to functionally express the H^+^/OC antiporter [21, 25, 26, 29, 35, 36], in the IPEC-J2 MDR1 cell line over-expressing the human version of P-gp, and lastly in wild type and mdr1a/1b (P-gp) knockout mice.

The rationale behind this study was to provide insights into the molecular mechanisms of triptan transport across the BBB with the long-term perspective of contributing to the central site of action hypothesis. This information can be helpful in the design of future anti-migraine compounds to be either more or less BBB penetrable. In addition, the results of this study could reveal potential transporter-related drug-drug interactions in migraine therapy. We demonstrate that the majority of the tested triptans inhibited H^+^/OC antiporter-mediated uptake of pyrilamine, with eletriptan emerging as the strongest inhibitor. Eletriptan, almotriptan, and sumatriptan translocated into hCMEC/D3 cells in a pH-dependent manner, but only eletriptan demonstrated saturable uptake kinetics in the tested concentration range. Eletriptan was moreover confirmed to be a substrate of P-gp. Neuropharmacokinetic assessment of eletriptan demonstrated low brain uptake in wild type mice, but with dominating brain uptake in mdr1a/1b knockouts. Overall, we propose that the putative H^+^/OC antiporter is an uptake pathway for eletriptan at the brain endothelium, and that other triptans also interact with the transporter.

## Methods

### Materials

All chemicals were purchased from Merck (Soeborg, Denmark) unless otherwise stated.

### Cell culture

The immortalized human cerebral microvascular endothelial cell line (hCMEC/D3) was maintained in T75-flasks coated with collagen type I from calf skin (C9791, 18.7 μg/mL) and cultured in EBM®-2 medium (Lonza, Basel, Schweiz) supplemented with 5% (v/v) fetal bovine serum (FBS, 10270, Life Technologies, CA, USA or SH3008803, ThermoFisher Scientific, MA, USA), 100 U/mL penicillin and 100 μg/ml streptomycin, 1 ng/mL human basic fibroblast growth factor (bFGF), 1.4 μM hydrocortisone, 1% (v/v) chemically defined lipid concentrate (ThermoFisher Scientific, MA, USA), 10 mM N-2-hydroxyethylpiperazine-N-2-ethane sulfonic acid (HEPES) and 5 μg/mL ascorbic acid (5% CO_2_, 37 °C). Culture media was changed every second or third day. Cells were passaged using trypsin–EDTA solution 10x (T4174, < 5 min, 5% CO_2_, 37 °C). For experiments, cells were seeded into 24-well plates (6.7·10^4^ cells/cm^2^) and cultured for four continuous days. The cells were used for experiments between passage 3–31.

The porcine ileum epithelial cell line IPEC-J2 transfected with human MDR1 [37] was maintained in T175-flasks and cultured in Dulbecco's Modified Eagle Medium/Nutrient Mixture F-12, supplemented with 10% (v/v) FBS (SH3008803, ThermoFisher Scientific, MA, USA), 100 U/mL penicillin and 100 μg/mL streptomycin, 2 mM L-glutamine, and 1 mM sodium pyruvate. The culture medium was supplemented with 2 µg/mL puromycin during maintenance. Culture media was changed every second or third day. Cells were passaged using trypsin–EDTA solution 10x (5–10 min, 5% CO_2_, 37 °C). For experiments, cells were either seeded into 24-well plates (1·10^5^ cells/cm^2^) and cultured for 2 days or seeded on Transwell® polyester inserts (3460, 1.12 cm^2^, pore size 0.4 µm) (3.57·10^4^ cells/cm^2^) and cultured for 15–17 days. IPEC-J2 MDR1 cells were used for experiments between passage 2–4.

### Cellular uptake studies

**Fig. 1:**
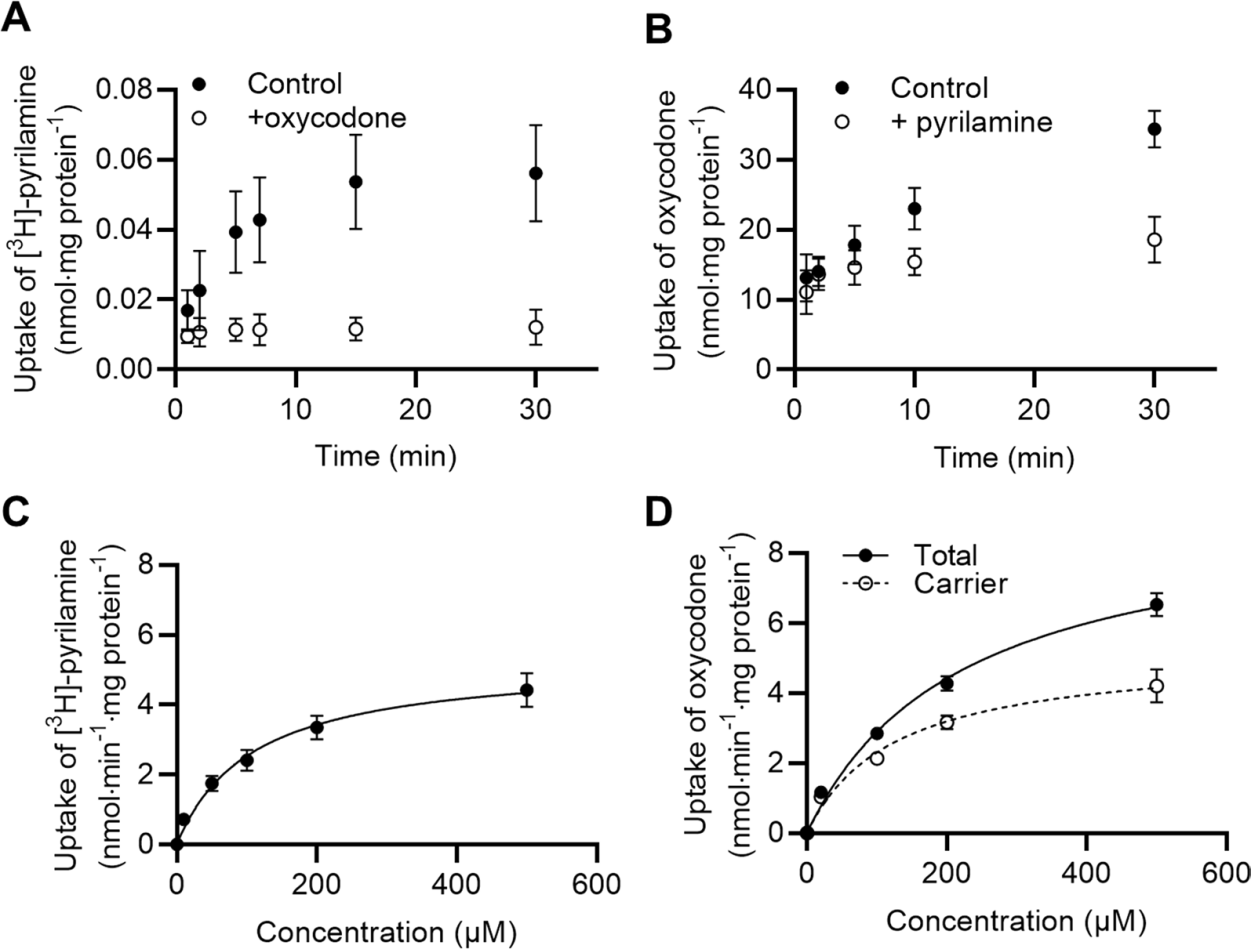
Uptake kinetics of prototypical H^+^/OC antiporter substrates into hCMEC/D3 cells. **A** Time-dependent uptake of [^3^H]-pyrilamine (1 µCi/mL, 48 nM) in the absence (Control) or presence of 500 µM oxycodone (+ oxycodone) (*n* = 2–3*,* ${{\text{N}}}_{total}=$ 6–9). **B** Time-dependent uptake of oxycodone (500 µM) in the absence (Control) or presence of 1000 µM pyrilamine (+ pyrilamine) (*n* = 3*, N*_*total*_ = *9*). **C** Concentration-dependent uptake of [^3^H]-pyrilamine (1 µCi/mL, 48 nM) in the presence of increasing concentrations of non-labelled pyrilamine. Data are fitted to non-linear regression (Michaelis–Menten kinetics) (*n* = 3*, N*_*total*_ = *9*). **D** Concentration-dependent uptake of oxycodone in the presence of 1000 µM pyrilamine. The total uptake is shown in closed circles, and the calculated carrier-mediated uptake (total—passive) is shown in open circles. Carrier-mediated uptake is fitted to non-linear regression (Michaelis–Menten kinetics) (*n* = 3*, N*_*total*_ = 9). All data points represent *mean* ± *SD*. Uptake amounts are normalized against protein content per well

The cells were washed twice with 37 °C Hank’s Balanced Salt Solution (HBSS) supplemented with 10 mM HEPES, 0.0375% (v/v) sodium bicarbonate and 0.05% (w/v) bovine serum albumin (BSA) and adjusted to pH 7.4 (hHBSS). The cells were equilibrated in hHBSS with or without inhibitor for 15 min (37 °C, 90 rpm). The compound of interest was spiked into the hHBSS followed by incubation for a designated time. The timeframe of the initial uptake rate was validated into hCMEC/D3 cells (Fig. 1 and Additional file 1). The experiment was terminated by washing three times with ice-cold HBSS supplemented with 0.0375% (v/v) sodium bicarbonate.

To assess the concentration-dependent uptake, the uptake flux was fitted to the Michaelis–Menten equation, Eq. 1.

1 $${{\text{J}}}_{{\text{ss}}}=\frac{{{\text{J}}}_{{\text{max}}} [{\text{S}}]}{{{\text{K}}}_{{\text{m}}}+[{\text{S}}]}$$

where J_ss_ represents flux under steady state, J_max_ is the maximal flux, [S] is the substrate concentration, and K_m_ is the Michaelis–Menten constant.

The inhibitory constant at 50% inhibition (IC_50_) was determined by fitting the uptake flux (J_ss_) as function of the logarithmic inhibitor concentration to Eq. 2.

2 $${{\text{J}}}_{{\text{ss}}}={{\text{J}}}_{{\text{min}}}+\frac{({{\text{J}}}_{{\text{max}}}-{{\text{J}}}_{{\text{min}}})}{1+{10}^{\left({\text{log}}\left({{\text{IC}}}_{50}\right)-\left[{\text{I}}\right]\right){{\text{n}}}_{{\text{H}}}}}$$

where [I] is the inhibitor concentration, n_H_ is the Hill coefficient.

The hCMEC/D3 cells were used for [^3^H]-pyrilamine inhibition studies between passage 3–6 (pyrilamine, oxycodone), 16–18 (almotriptan), 7–13 (eletriptan), 25–27 (frovatriptan), 3–8 (naratriptan), 16–18 (rizatriptan), 12–14 (sumatriptan), and 14–16 (zolmitriptan).

The hCMEC/D3 cells were used for time-dependent uptake studies between passage 3–5 (pyrilamine), 7–9 (oxycodone), 6–8 (almotriptan), and 24–31 (eletriptan).

The hCMEC/D3 cells were used for concentration-dependent uptake studies between passage 21–23 (pyrilamine), 11–13 (oxycodone), 12–14 (almotriptan), 16–18 (eletriptan), and 12–14 (sumatriptan).

The hCMEC/D3 cells were used for uptake studies of almotriptan, eletriptan, and sumatriptan in the presence of pyrilamine and oxycodone between passage 21–23.

The IPEC-J2-MDR1 cells were used for [^3^H]-digoxin inhibition studies between passage 2–4.

### Manipulation of intra- and extracellular pH

Intracellular pH was manipulated as earlier described by Byron et al. [38] to assess the involvement of a proton antiporter, i.e. the H^+^/OC antiporter. Briefly, elevated intracellular pH during cellular uptake was achieved by adding the substrate of interests immediately (< 10 s) after exposure to 30 mM NH_4_Cl in hHBSS. Decreased intracellular pH during cellular uptake was achieved by preincubation with 30 mM NH_4_Cl in hHBSS (30 min, 37 °C) followed by aspiration and addition of hHBSS buffer. After approximately 60 s, the compound of interest was added to the buffer. Extracellular pH manipulation was achieved by adding hHBSS adjusted to pH 6.8, 7.4, or 8.2 during cellular uptake. The hCMEC/D3 cells were used for pH manipulation experiments between passage 17–19 (pyrilamine), 10–12 (oxycodone, almotriptan), 12–14 (eletriptan), and 3–5 (sumatriptan).

### Intracellular pH measurements

Intracellular pH changes were assessed with the fluorescent pH indicator 2′,7′- bis-(Carboxyethyl)-5(6′)-carboxyfluorescein acetoxymethyl ester (BCECF-AM) using a NOVOstar plate reader (BMG TABTECH GmbH, Offenburg, Germany). Fluorescence intensities were measured using a dual excitation of 485 and 380 nm and an emission filter of 520 nm. Briefly, hCMEC/D3 cells were seeded into a 96-well plate (3·10^4^ cells/cm^2^) and cultured for three continuous days (5% CO_2_, 37 °C). Cell media was changed the day before an experiment. A 5 µM BCECF-AM loading solution was prepared in hHBSS without BSA (hHBSS(-)) followed by sonication using a tip sonicator S-4000 (60 amplitude, 120 s, Misonix, NY, USA), to boost dissolution, and equilibrated to 37 °C. The cells were washed twice with 37 °C hHBSS(-) and loaded with the BCECF-AM solution (45 min, protected from light, 37 °C). The cells were washed three times with 37 °C hHBSS(-) and loaded with either hHBSS(-) or 30 mM NH_4_Cl in hHBSS(-). Control cells were preincubated with hHBSS(-) followed by automated injection of hHBSS(-) to validate the influence of injection on the fluorescence signal. Cells exposed to acute NH_4_Cl were preincubated with hHBSS(-) followed by automated injection of NH_4_Cl to a final concentration of 30 mM. For automated injections during measurements, the injection volume was 33 µL with a speed of 310 µL/s. Cells exposed to NH_4_Cl preincubation were preincubated with 30 mM NH_4_Cl in hHBSS(-) for 30–60 min followed by measurements of the fluorescent baseline. The NH_4_Cl solution was manually aspirated followed by manual loading of 100 µL hHBSS(-). Immediately thereafter, the fluorescence was assessed. The fluorescent signal was normalized against baseline signals. The hCMEC/D3 cells were used for intracellular pH measurements between passage 26–28.

### Barrier integrity measurements

The barrier integrity of the IPEC-J2 MDR1 cell monolayers cultured on semipermeable supports was assessed by transepithelial electrical resistance (TEER) measurements. Cells were allowed to equilibrate at room temperature for 20 min. The resistance was measured using an Endohm 12-cup electrode chamber (World Precision Instruments Inc., FL, USA) connected to an EVOM voltmeter (World Precision Instruments Inc., FL, USA). Measured TEER values were subtracted by the TEER value of a blank filter (14 Ω), normalized to the surface area of the permeable support (1.12 cm^2^), and expressed as Ω·cm^2^. The IPEC-J2 MDR1 cells were used for barrier integrity measurements between passage 2–4.

### Bidirectional transport

Bidirectional transport studies were conducted using IPEC-J2 MDR1 cell monolayers cultured on semipermeable Transwell® polyester inserts (Corning Inc., NY, USA, 3460, 1.12 cm^2^, pore size 0.4 µm). The cells were washed twice with 37 °C hHBSS. Before initiation of the experiment, the cells were equilibrated in 37 °C hHBSS with or without zosuquidar (ZSQ) (2 µM) for 15 min (37 °C, 90 rpm). After equilibration, eletriptan HBr (50 µM) was spiked into the donor compartment. Samples were taken from the receiver compartment after 15, 30, 45, 60, 90, and 120 min. Samples of 100 uL were withdrawn from the basolateral compartment, while samples of 50 µL were withdrawn from the apical compartment. The withdrawn sample volume was immediately replaced with an equal volume of 37 °C hHBSS with or without ZSQ. Donor samples were sampled at the end of the experiment. The IPEC-J2 MDR1 cells were used in bidirectional transport experiments between passage 2–4.

The accumulated amount of drug (Q) was calculated for each time-point using Eq. 3.

3 $$Q={V}_{s}\left(\sum_{n-1}^{n}{C}_{n-1}\right)+{C}_{n}{V}_{t}$$

where V_s_ represents the sample volume, V_t_ the total volume of the receiver compartment, C_n_ is the concentration of the sample n.

Steady state flux (J_steady state_) was calculated as the slope of the linear part of the accumulated amount (Q) in the receiver compartment as function of time from Eq. 4.

4 $${J}_{ss}=\frac{\Delta Q}{\Delta t \cdot A}$$

where t represents time and A is the area of the permeable support.

The apparent permeability (P_app_) was calculated from Eq. 5.

5 $${P}_{app}=\frac{{J}_{ss}}{{C}_{donor}}$$

where C_donor_ represents the initial concentration in the donor compartment.

Efflux ratios (ER) were calculated as the ratio of the apparent permeability in the basolateral to apical direction P_app_(B-A) and the permeability in the apical to basolateral direction P_app_(A-B) using Eq. 6.

6 $${\text{ER}}=\frac{{{\text{P}}}_{{\text{app}}}\left({\text{B}}-{\text{A}}\right)}{{{\text{P}}}_{{\text{app}}}({\text{A}}-{\text{B}})}$$

### Sample preparation and quantification of radiolabeled compounds from in vitro experiments

The cells were permeabilized using 0.1% (v/v) Triton X-100 in ultrapure water (ELGA, Buckinghamshire, England) for 10 min at room temperature. Thereafter, the cells were scraped off the bottom of the wells, transferred to scintillation vials, and mixed with 2 mL of Ultima Gold 241 TM (Perkin Elmer, MA, USA). The samples were analyzed using a Tri-Carb 2910 TR Liquid Scintillation Analyzer (Perkin Elmer, MA, USA).

### Sample preparation and quantification of unlabeled compounds from in vitro experiments

The cells were permeabilized using 50% (v/v) acetonitrile (HPLC LC–MS grade, VWR International S.A.S, Soeborg, Denmark) in ultrapure water (ELGA, Buckinghamshire, England) for 10 min at room temperature. Thereafter, the cells were scraped off the bottom of the wells and transferred to HPLC vials. Samples from bidirectional transport experiments were transferred directly into HPLC vials. All samples were stored at – 18 °C for later analysis. Calibration curves with standards in the range of 1 or 5–1000 ng/mL were dissolved in 50% (v/v) acetonitrile in ultrapure water (ELGA, Buckinghamshire, England), or in hHBSS(-) for analysis of bidirectional transport samples.

Liquid chromatography tandem mass spectrometry (LC–MS/MS) were performed using electrospray ionization in positive mode on a micromass Quattro micro™ API tandem quadrupole mass spectrometer (Waters, MA, USA) or an Ultivo triple quadrupole mass spectrometer (Agilent Technologies, CA, USA) coupled to Agilent HPLC system 1100 Series or 1260 Series (Agilent Technologies, CA, USA California), respectively. Applied columns were an InfinityLab Poroshell 120 EC-C18 (3.0 × 50 mm; 2.7 micron, Agilent Technologies, CA, USA) or a Kinetex 2.6 μm XB-C18 (100 Å, 100 × 4.6 mm, Phenomenex Inc., CA, USA). An overview of applied HPLC and MS/MS parameters can be found in Additional file 2 and Additional file 3, respectively. The data acquisition software was MassLynx (V4.1, Waters, MA, USA).

### Quantification of total protein content

The protein content of hCMEC/D3 cells was determined for normalization purposes. Cells were washed with ice-cold phosphate buffered saline following cell lysis in cell extraction buffer (FNN0011, Thermo Fisher Scientific, MA, USA) supplemented with 1 × cOmplete™ Protease Inhibitor Cocktail (04693116001, Roche, Basel, Schweiz), 1 mM phenylmethylsulfonyl fluoride, and 1 mg/mL pepstatin for 30 min on ice. The protein concentration was determined using the bicinchoninic acid (BCA) assay kit (bicinchoninic acid and copper (II) sulphate solution) as directed by the manufacturer. Samples were measured using a SPECTROstar Nano microplate reader (BMG LABTECH, Ortenberg, Germany).

### Animals

Male Abcb1a/Abcb1b-eKO1 (P-gp knockout) mice and FVB wild-type counterparts (18–24 g at arrival) were obtained from Shanghai Biomodel Organism Science & Technology Development Co. Ltd (Shanghai, China). Male C57 mice (C57BL/6N) (18–22 g at arrival) were obtained from Beijing Vitalstar Biotechnology Co., Ltd (Beijing, China). Animals were housed in pairs in a temperature-controlled environment (20–24 °C) with lighting maintained under a 12-h light–dark cycle. Animals were habituated for at least seven days prior to surgery with free access to food and water. The experiments were carried out in accordance with the Danish legislation regulating animal experiments; Law and Order on Animal experiments; Act No. 1107 of 01/07/2022 and Act No. 1108 of 01/07/2022 and with the specific license for this experiment issued by the National Authority.

### In vivo brain distribution by neuropharmacokinetic assessment and equilibrium dialysis

In vivo experiments were conducted to assess the extent of the BBB transport of eletriptan and to elucidate the involvement of active transport processes. All mice were cannulated in the jugular vein for intravenous drug administration. Following surgery, the animals were allowed to recover for 3 days before administration of test compounds. Eletriptan HBr was dosed in P-gp knockout and wild type mice using a loading dose of 2 mg/kg (as bolus) followed by a 2-h constant rate infusion of 3 mg/kg (1.5 mg/kg/h) dissolved in 0.9% NaCl. Diphenhydramine hydrochloride was dosed in C57 mice using a 2-h constant rate infusion of 5 mg/kg (2.5 mg/kg/h) dissolved in 10% HP-β-CD in water. Throughout all studies a dose volume of 10 mL/kg was applied. Serial blood samples were taken from the saphenous vein during the infusion to verify steady state and at the end of infusion. Terminal blood samples were collected from all animals. Brain samples were prepared by homogenization using ultrasonication as described previously [39] and analyzed together with the plasma samples using LC–MS/MS [39]. The lower limit of quantification was determined to 1.0 ng/mL in plasma and 5 ng/g in brain tissue for both eletriptan and diphenhydramine.

Plasma protein and brain tissue binding of eletriptan and diphenhydramine were determined in vitro by equilibrium dialysis using donor test compound solutions of 1 µM incubated in triplicate as described previously [40]. C57 mice were used to prepare blank plasma and brain matrices throughout all binding studies.

The total brain and plasma concentration partition coefficient (K_p_) was calculated from Eq. 7.

7 $${{\text{K}}}_{{\text{p}}}=\frac{{{\text{C}}}_{{\text{tot}},{\text{brain}},{\text{ss}}} }{{{\text{C}}}_{{\text{tot}},{\text{plasma}},{\text{ss}}}}$$

where C_tot,brain,ss_ and C_tot,plasma,ss_ is the total drug concentration at steady state in brain and plasma, respectively.

The unbound brain and plasma partition coefficient (K_p,uu_) was derived from dividing the unbound brain concentration with the unbound plasma concentration at the end of infusion for each animal. The K_p,uu_ was calculated from Eq. 8.

8 $${{\text{K}}}_{{\text{p}},{\text{uu}}}=\frac{{{\text{C}}}_{{\text{tot}},\mathrm{ brain},{\text{ss}}} \cdot {{\text{f}}}_{{\text{u}},{\text{brain}}}}{{{\text{C}}}_{{\text{tot}},{\text{plasma}},{\text{ss}}} \cdot {{\text{f}}}_{{\text{u}},{\text{plasma}}}}$$

where f_u,brain_ and f_u,plasma_ is the fraction of unbound drug in the brain and plasma, respectively.

### Statistical analysis

Statistical analysis was conducted using GraphPad Prism version 9.4.0 (La Jolla, California, USA). Data are presented as mean ± standard deviation (SD) unless otherwise stated. In vitro experiments were performed in three biological replicates and three technical replicates, unless otherwise stated, where *n* denotes the number of biological replicates and *N*_*total*_ denotes the total number of technical replicates. In vivo experiments were performed in groups of three animals. Statistical analyses were performed by comparing the group means with either two-tailed unpaired Students t-test for comparing two means or by one-way analysis of variance (ANOVA) followed by a Tukey’s multiple comparison test for comparison of more than two means. P-values < 0.05 were considered statistically significant.

## Results

### Physicochemical characteristics of triptans and prototypical H^+^/OC antiporter substrates

Triptans are a relatively homogeneous group of drug compounds with similar physicochemical properties, including molecular weights < 400 g/mol and a low to moderate lipophilicity (cLogD values ranging from − 2.1–0.18) [41]. Triptans either possess a secondary or a tertiary amine with pK_a_ values ranging from 8.4 to 10.4, rendering members of this drug class cationic at physiological pH. As a consequence, it is unlikely that triptans cross the brain microvascular endothelium through passive diffusion to a clinically relevant extent. Table 1 summarizes structures and physicochemical properties of the triptans, as well as oxycodone and pyrilamine, which are two prototypical H^+^/OC antiporter substrates included in this study.

**Table 1:** Structures and physicochemical properties of almotriptan, eletriptan, frovatriptan, naratriptan, rizatriptan, sumatriptan, zolmitriptan, oxycodone, and pyrilamine

Compound	Structure pH 7.4	Strongest basic pK_a_^a^	Overall charge pH 7.4	Mw (g/mol)^b^	cLogD pH 7.4^c^
Almotriptan		9.6	Positive	335.5	0.3
Eletriptan		8.4	Positive	382.5	1.1
Frovatriptan		10.4	Positive	243.3	− 1.5
Naratriptan		9.2	Positive	335.5	− 0.2
Rizatriptan		9.6	Positive	269.3	0.04
Sumatriptan		9.5	Positive	295.4	− 0.6
Zolmitriptan		9.6	Positive	287.4	0.4
Oxycodone		8.8	Positive	315.4	0.5
Pyrilamine		8.8	Positive	285.4	1.1

The secondary or tertiary amine is marked in red

^a^Extracted from DrugBank® [42]

^b^Extracted from PubChem [43]

^c^Extracted from ChemSpider [44]

### The H^+^/OC antiporter was functionally expressed in the hCMEC/D3 cells

The functionality of the H^+^/OC antiporter was probed in the hCMEC/D3 cell line using the prototypical substrates [^3^H]-pyrilamine and oxycodone. The following criteria were used to confirm functional expression of the H^+^/OC antiporter: (1) Saturable uptake kinetics, (2) competitive inhibition between prototypical H^+^/OC antiporter substrates, and (3) pH-dependent uptake of substrates.

For both [^3^H]-pyrilamine (Fig. 1A) and oxycodone (Fig. 1B), the time-dependent uptake approached a plateau within a 30-min timeframe. The uptake of [^3^H]-pyrilamine and oxycodone was reduced by the presence of 500 µM oxycodone and 1000 µM pyrilamine, respectively. We observed concentration-dependent uptake of [^3^H]-pyrilamine and oxycodone, both revealing saturable uptake kinetics (Fig. 1C and D). The saturable uptake kinetics indicate that the transport is driven by an active uptake mechanism.

The estimated kinetic parameters for [^3^H]-pyrilamine and oxycodone uptake into the hCMEC/D3 cells are presented in Table 2. The data indicate a slightly higher transporter affinity of pyrilamine (K_m_ value of ~ 93 µM) as compared to oxycodone (K_m_ value of ~ 130 µM). No differences were observed in the maximal uptake rate (J_max_).

**Table 2:** Kinetic uptake parameters of prototypical H^+^/OC antiporter substrates, pyrilamine and oxycodone, into hCMEC/D3 cells

Compound	Apparent K_m_ (µM)	J_max_ (nmol·min^−1^·mg protein^−1^)
Pyrilamine	93 ± 17	5.1 ± 0.7
Oxycodone	130 ± 57	5.3 ± 1.0

Kinetic parameters are represented as *mean* ± *SD* (*n* = 3*, N*_*total*_ = 9)

The putative H^+^/OC antiporter has been described to function as a proton/drug antiporter [19, 20]. To validate the proton-dependency, the cellular uptake of [^3^H]-pyrilamine and oxycodone was probed under conditions, where the transmembrane proton gradient was altered by manipulating the intracellular or extracellular pH. Briefly, the uptake of both compounds was affected by manipulating the intracellular and extracellular pH supporting the evidence that these compounds are transported via a proton-driven mechanism, refer Additional file 4**.** Intracellular alkalization or extracellular acidification resulted in a reduced cellular uptake, while intracellular acidification or extracellular alkalization resulted in an increased cellular uptake, both indicating an oppositely directed proton driving force. Confirmation of intracellular pH changes by NH_4_Cl exposure is shown in Additional file 5.

To summarize, the hCMEC/D3 cells revealed characteristics supporting functional expression of the putative H^+/^OC antiporter including saturable uptake kinetics, competitive uptake inhibition, and pH-dependent uptake of known H^+^/OC antiporter substrates. Therefore, the hCMEC/D3 cells were considered a suitable model to investigate the involvement of the H^+^/OC antiporter in brain endothelial cell uptake of triptans.

### Triptans demonstrated inhibition of [^3^H]-pyrilamine uptake with IC_50_ values ranging from 15 to 1729 µM

**Fig. 2:**
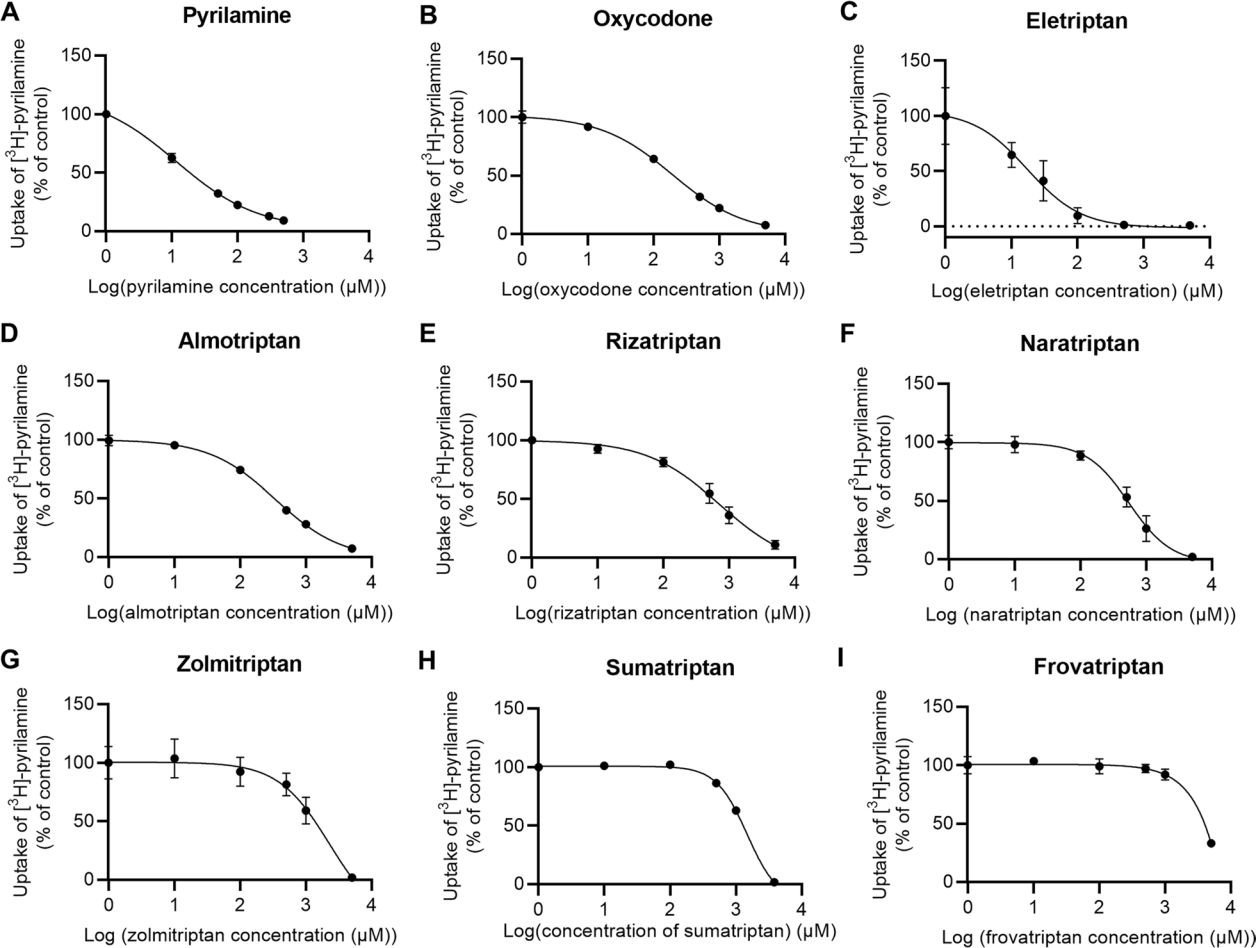
Concentration-dependent inhibition of [^3^H]-pyrilamine uptake into hCMEC/D3 cells. Concentration-dependent inhibition of [^3^H]-pyrilamine (1 µCi/mL, 48 nM) in the presence of **A** pyrilamine, **B** oxycodone, **C** eletriptan, **D** almotriptan, **E** rizatriptan, **F** naratriptan, **G** zolmitriptan, **H** sumatriptan, and **I** frovatriptan. Note that the logarithm to 0 µM (control) is designated 0 in the figure. Data are fitted to non-linear regression (log(inhibitor) vs. response—Variable slope (four parameters)) (*n* = 2–3*, N*_*total*_ = 6–9). All data points represent *mean* ± *SD*

To assess the involvement of the H^+^/OC antiporter in the uptake of triptans into endothelial cells, we determined the cellular uptake of the prototypical H^+^/OC antiporter substrate, [^3^H]-pyrilamine (48 nM), in the presence of increasing concentrations of the seven different triptans (Fig. 2). We included the H^+^/OC antiporter substrates, non-labelled pyrilamine and oxycodone, as positive controls for uptake inhibition (Fig. 2A and B). The apparent IC_50_ values are presented in Table 3.

**Table 3:** Apparent IC_50_ values on [^3^H]-pyrilamine uptake (1 µCi/mL, 48 nM) into hCMEC/D3 cells

Compound	Apparent IC_50_ (µM)
Pyrilamine	11 ± 6
Oxycodone	225 ± 57
Eletriptan	15 ± 4
Almotriptan	305 ± 83
Naratriptan	460 ± 41
Rizatriptan	719 ± 243
Sumatriptan	1458 ± 189
Zolmitriptan	1729 ± 1209
Frovatriptan	N/A

The IC_50_ values represent *mean* ± *SD* (*n* = 2–3*, N*_*total*_ = 6–9)

The most prominent triptan-induced inhibition of pyrilamine was observed in the presence of eletriptan (IC_50_ value of ~ 15 µM) **(**Fig. 2C**)**, which was comparable to pyrilamine self-inhibition (IC_50_ of ~ 11 µM). Almotriptan exhibited the second most pronounced inhibition of pyrilamine (IC_50_ value of ~ 305 µM) (Fig. 2D), a potency comparable to that of oxycodone (IC_50_ value of ~ 225 µM). The remaining triptans inhibited the uptake of pyrilamine in the order of naratriptan > rizatriptan > sumatriptan > zolmitriptan > frovatriptan **(**Fig. 2E-I). An IC_50_ value could not be obtained for frovatriptan as it did not exhibit sufficient inhibition in the investigated concentration range.

### Eletriptan was taken up by hCMEC/D3 cells in a saturable manner

Our inhibition studies indicated that the majority of the triptans competed with pyrilamine for the H^+^/OC antiporter binding site but did not reveal whether they are actual substrates being translocated by the transporter. Three triptans, eletriptan, almotriptan, and sumatriptan, were evaluated as to whether they were transported via the H^+^/OC antiporter in uptake studies using hCMEC/D3 cells and LC–MS/MS for substrate detection. Eletriptan and almotriptan were selected based on their high inhibitory affinity, while sumatriptan was selected based on its poor inhibitory properties.

**Fig. 3:**
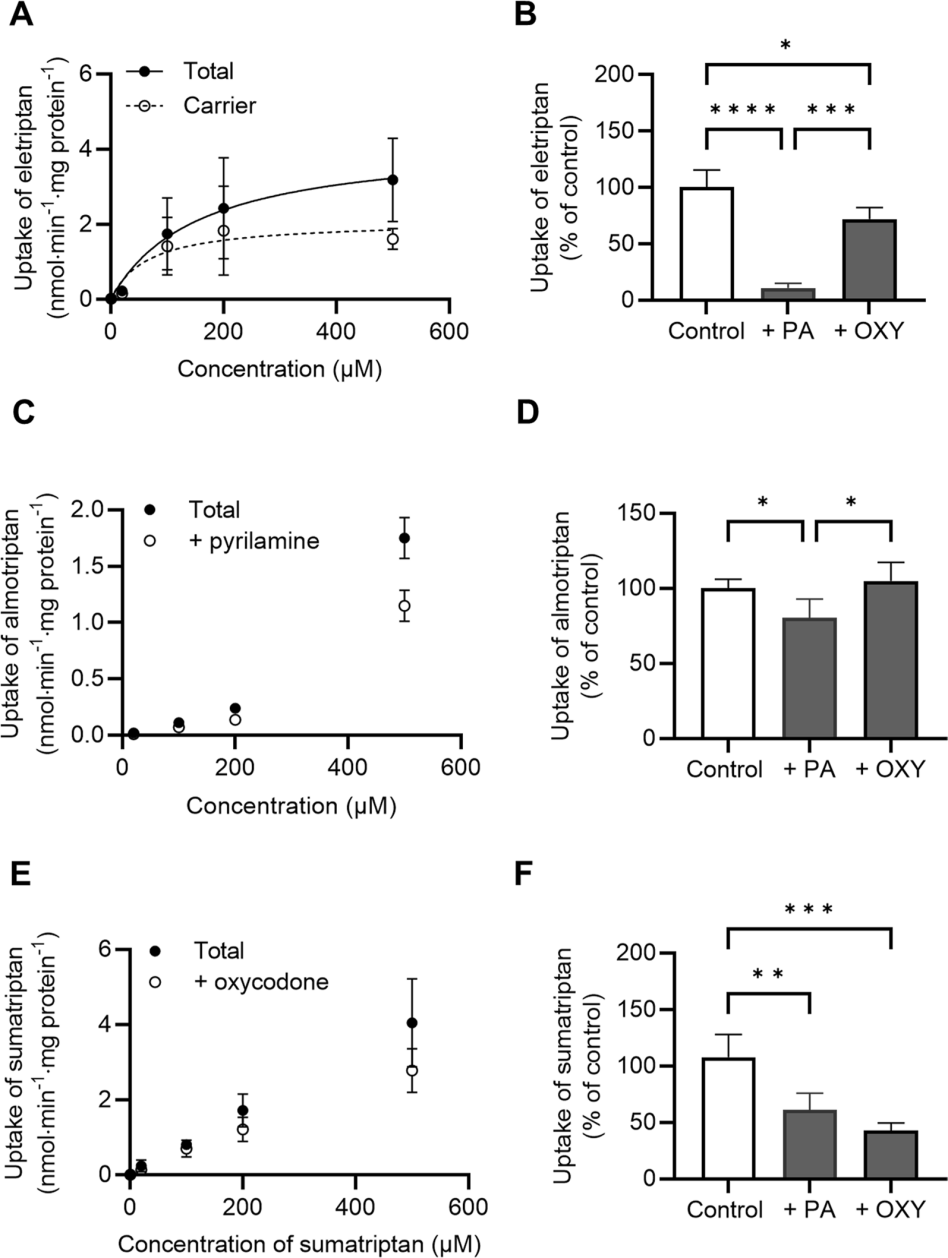
Uptake properties of eletriptan, almotriptan and sumatriptan into hCMEC/D3 cells. **A** Concentration-dependent uptake of eletriptan in the presence of 1000 µM pyrilamine. The total uptake is shown in closed circles, and the calculated carrier-mediated uptake (total–passive) is shown in open circles. Carrier-mediated uptake is fitted to non-linear regression (Michaelis–Menten kinetics). **B** Uptake of eletriptan (50 µM) in absence (Control) or presence of 500 µM pyrilamine (PA) or 500 µM oxycodone (OXY). **C** Concentration-dependent uptake of almotriptan in the presence of 1000 µM pyrilamine (+ pyrilamine). **D** Uptake of almotriptan (50 uM) in absence (Control) or presence of 500 µM pyrilamine (PA) or 500 µM oxycodone (OXY). **E** Concentration-dependent uptake of sumatriptan in the presence of 1000 µM oxycodone (+ oxycodone). **F** Uptake of sumatriptan (50 uM) in absence (Control) or presence of 500 µM pyrilamine (PA) or 500 µM oxycodone (OXY). All data points or columns represent *mean* ± *SD* (*n* = 3*, N*_*total*_ = 9). Uptake amounts are normalized against protein content per well. Data were analysed using a one-way ANOVA followed by a Tukey’s multiple comparison test. *: P ≤ 0.05. **: P ≤ 0.01. ***: P ≤ 0.001. ****: P ≤ 0.0001

Eletriptan showed concentration-dependent uptake into hCMEC/D3 cells reaching saturation within a 500 µM concentration (Fig. 3A, Table 4), indicative of carrier-mediated uptake**.** The H^+^/OC antiporter substrates pyrilamine and oxycodone inhibited the uptake of eletriptan in a statistically significant manner, with the most pronounced inhibition observed in the presence of pyrilamine **(**Fig. 3B**)**. Almotriptan did not show saturable uptake, but uptake was slightly inhibited in the presence of pyrilamine (Fig. 3C and D). Oxycodone did not inhibit the uptake of almotriptan (Fig. 3D). The uptake of sumatriptan showed a linear increase as function of increasing concentrations (Fig. 3E). Both pyrilamine and oxycodone inhibited the uptake of sumatriptan, with the most pronounced inhibition observed in the presence of oxycodone (Fig. 3F).

**Table 4:** Kinetic uptake parameters of eletriptan, almotriptan, and sumatriptan into hCMEC/D3 cells

Compound	Apparent K_m_ (µM)	J_max_ (nmol·min^−1^·mg protein^−1^)
Eletriptan	89 ± 38	2.2 ± 0.7
Almotriptan	N/A	N/A
Sumatriptan	N/A	N/A

Kinetic parameters are represented as *mean* ± *SD* (*n* = 3*, N*_*total*_ = 9)

### Uptake of eletriptan, almotriptan, and sumatriptan in hCMEC/D3 cells were affected by the transmembrane proton gradient

**Fig. 4:**
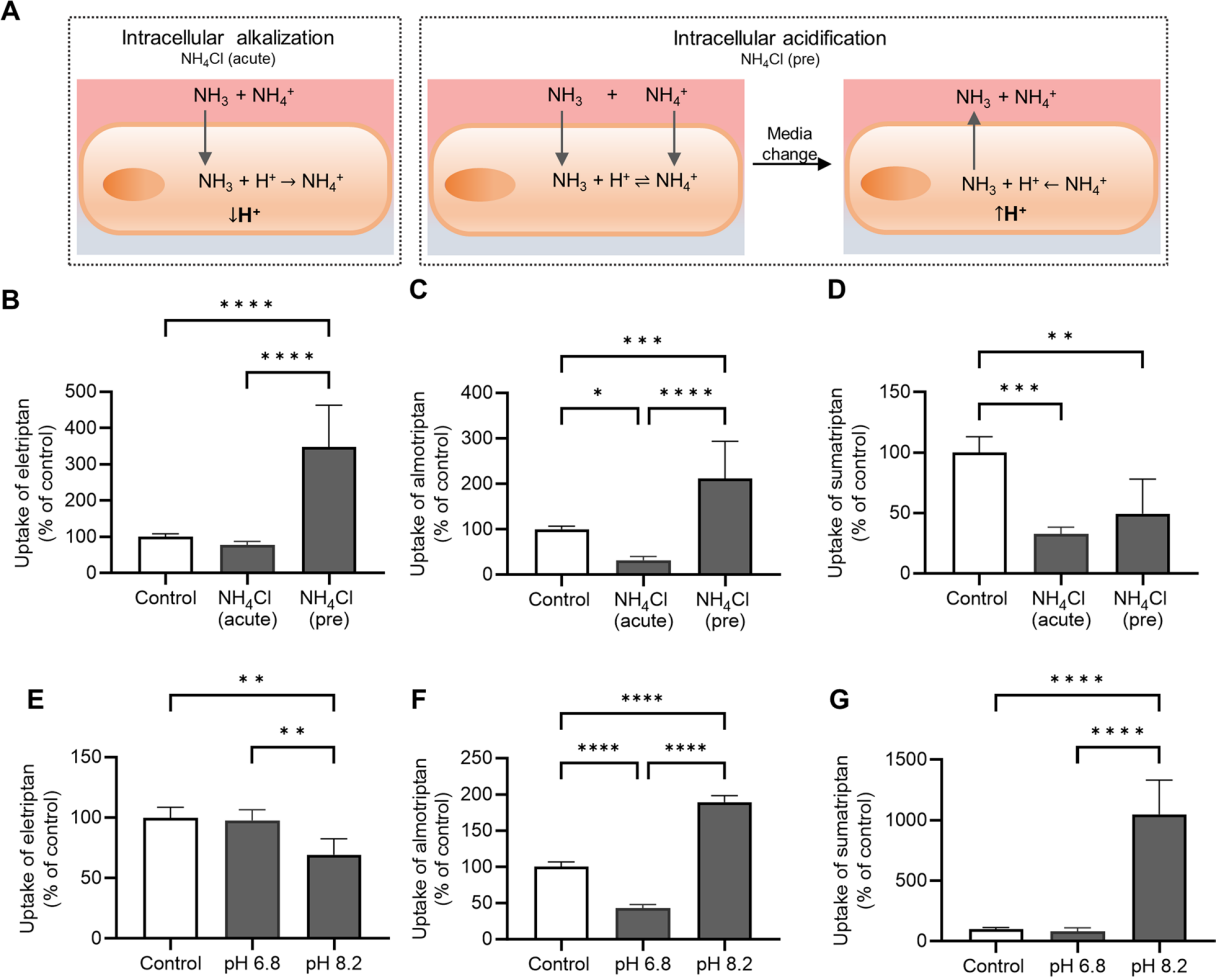
Cellular uptake of eletriptan, almotriptan and sumatriptan after intra- and extracellular pH manipulation in hCMEC/D3 cells. **A** Schematic representation of intracellular pH changes after acute- or preexposure to 30 mM NH_4_Cl. Uncharged NH_3_ diffuses into the cells and consumes intracellular protons, consequently leading to intracellular alkalization. Intracellular acidification will occur upon preexposure to NH_4_Cl followed by media change. After 30 min of preexposure to NH_4_Cl, both NH_3_ and NH_4_^+^ will enter the cells and reach equilibrium. Upon media change, uncharged NH_3_ will quickly diffuse out of the cells causing intracellular accumulation of protons, which will lead to intracellular acidification. **B-D)** Uptake of triptans (500 µM) was investigated in transport buffer pH 7.4 (Control), after acute exposure to NH_4_Cl (NH_4_Cl (acute)), and after preexposure to NH_4_Cl followed by media change (NH_4_Cl (pre)). Effect of intracellular pH manipulation on **B** eletriptan uptake, **C** almotriptan uptake, and **D** sumatriptan uptake. **E–G** Uptake of triptans (500 µM) was investigated in transport buffer with pH 7.4 (Control), pH 6.8 and pH 8.2. Effect on extracellular pH manipulation on **E** eletriptan uptake **F** almotriptan uptake, and **G** sumatriptan uptake. Each column represents *mean* ± *SD* (*n* = 3*, N*_*total*_ = 9). Data were analysed using a one-way ANOVA followed by a Tukey’s multiple comparison test. *:P ≤ 0.05. **: P ≤ 0.01. ***: P ≤ 0.001. ****: P ≤ 0.0001

It was evaluated whether eletriptan, almotriptan, and sumatriptan were taken up by hCMEC/D3 cells in a proton-dependent manner, as would be expected from H^+^/OC antiporter-mediated transport. Cellular uptake of the triptans were performed under conditions where the transmembrane proton gradient was altered through manipulation of the extra- or intracellular pH by acute- or preexposure to 30 mM NH_4_Cl (Fig. 4A). The resulting intracellular alkalization or acidification after acute or preexposure to 30 mM NH_4_Cl was determined using the fluorescent intracellular probe BCECF, as documented in Additional file 5.

Cellular uptake of eletriptan, almotriptan, and sumatriptan was decreased after intracellular alkalization, caused by acute exposure of the hCMEC/D3 cells to 30 mM NH_4_Cl (Fig. 4B-D). Intracellular acidification, caused by pre-exposure to NH_4_Cl followed by media change, resulted in a statistically significant increase in the uptake of eletriptan and almotriptan but a decreased uptake of sumatriptan (Fig. 4B–D). Unexpectedly, extracellular acidification (pH 6.8) did not affect eletriptan uptake whereas a slight decrease in eletriptan uptake was observed at pH 8.2 (Fig. 4E). The uptake of both almotriptan (Fig. 4F) and sumatriptan (Fig. 4G) was reduced at pH 6.8 and increased at pH 8.2.

In summary, the uptake of eletriptan, sumatriptan, and almotriptan all showed transmembrane proton gradient dependency in a manner consistent with uptake via a proton antiporter.

### Eletriptan displayed P-gp substrate characteristics, i.e. inhibition of digoxin efflux and polarized B-A transport in the IPEC-J2 MDR1 cell line

**Fig. 5:**
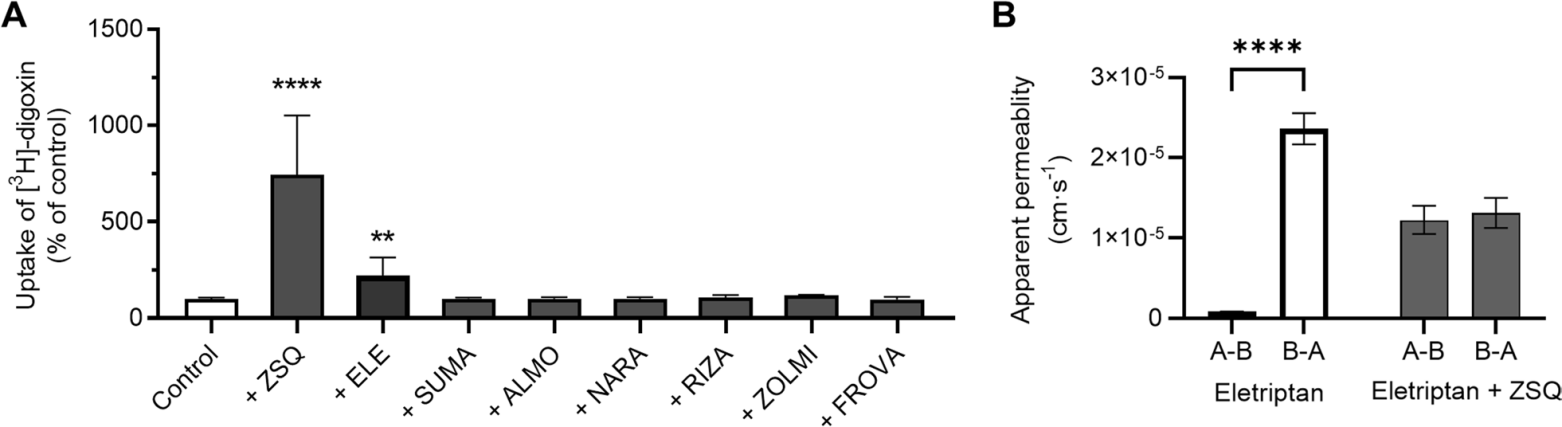
Triptans interaction with P-gp in IPEC-J2 MDR1 cells. **A** Screening of the inhibitory effects of triptans on [^3^H]-digoxin uptake into IPEC-MDR1 cells. Uptake of [^3^H]-digoxin (1 µCi/mL, 42 nM) for 15 min in the absence (Control) or presence of 2 µM zosuquidar (ZSQ), 500 µM eletriptan (ELE), 500 µM almotriptan (ALMO), 500 µM rizatriptan (RIZA), 500 µM zolmitriptan (ZOLMI), 500 µM naratriptan (NARA), 500 µM sumatriptan (SUMA) or 500 µM frovatriptan (FROVA). Each column represents *mean* ± *SD* (*n* = 3*, N*_*total*_ = 9). Columns were compared to’Control-group’ and analysed using a student’s t-test. **:P ≤ 0.01. ****: P ≤ 0.0001. **B** Apparent permeabilities (Papp) of eletriptan (50 µM) in the absence or presence of ZSQ (2 µM) in the A-B or B-A direction. Each column represents *mean* ± *SD* (*n* = 3*, N*_*total*_ = 9). Data were analysed using a two-way ANOVA followed by a Tukey’s multiple comparison test. ****: P ≤ 0.0001

Our aim was to investigate the mechanisms underlying transport mechanisms of triptans at the BBB. Having established that at least eletriptan showed characteristics of carrier-mediated transport via the H^+^/OC antiporter, we continued to examine whether there was an interaction between the triptans and P-gp, which could potentially lead to triptans being unable to pass the BBB [45, 46]. We screened for potential interactions between the triptans and the prototypical P-gp substrate digoxin using the IPEC-J2 MDR1 cell line [37, 47]. The cellular uptake of [^3^H]-digoxin (42 nM), was investigated in the absence or presence of eletriptan, sumatriptan, almotriptan, naratriptan, rizatriptan, zolmitriptan, or frovatriptan (500 µM) **(**Fig. 5A**)**. The specific P-gp inhibitor ZSQ (2 µM) was used as positive control for P-gp inhibition. [^3^H]-digoxin uptake was increased in a statistically significant manner in the presence of ZSQ (742 ± 308% of control). Likewise, the presence of eletriptan resulted in a statistically significant increase of [^3^H]-digoxin uptake (222 ± 92% of control), indicating that eletriptan inhibited the P-gp-mediated efflux of [^3^H]-digoxin. Eletriptan was the only triptan which had an impact on [^3^H]-digoxin uptake in the IPEC-J2 MDR1 cells.

To confirm the notion that eletriptan might be a P-gp substrate, we performed a bidirectional transport study with eletriptan (50 µM) across IPEC-J2 MDR1 monolayers in the absence or presence of ZSQ (2 µM). The TEER across the IPEC-J2 MDR1 monolayers was 1580 ± 95 Ω·cm^2^ (*n* = 3, *N*_*total*_ = 36) prior to the experiments. The apparent permeability (Papp) of eletriptan was statistically significant higher in the B to A direction (2.4 ± 0.2 ·10^–5^ cm·s^−1^) than in the A to B direction (8.2 ± 0.5 ·10^–7^ cm·s^−1^) (Fig. 5B). Flux curves are shown in Additional file 6. The efflux ratio (ER) for eletriptan was calculated to be 28.9 ± 1.8, thus demonstrating active efflux. The polarized bidirectional transport of eletriptan was completely abolished in the presence of ZSQ (Papp_A-B_ 1.2 ± 0.2 10^–5^ cm·s^−1^ and Papp_B-A_ 1.3 ± 0.2 10^–5^ cm·s^−1^), which reduced the ER close to unity (1.1 ± 0.1). These results further show that eletriptan is a substrate of the P-gp efflux pump.

### Eletriptan brain-to-plasma distribution in wild type and mdr1a/1b knockout mice indicated that active carrier-mediated uptake and P-gp-mediated efflux occurs simultaneously at the BBB

**Fig. 6:**
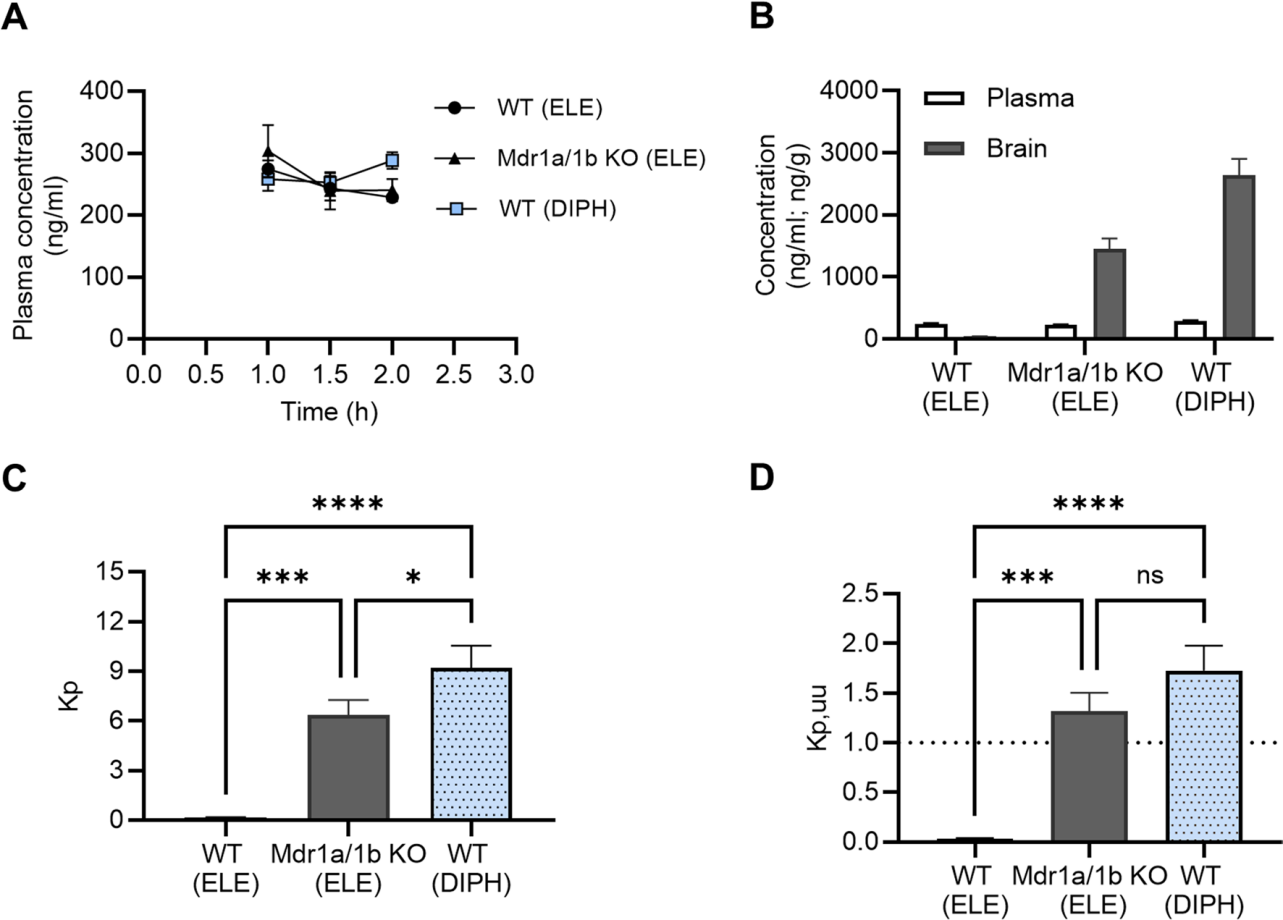
Neuropharmacokinetic assessment of eletriptan and diphenhydramine in wild type (WT) and mdr1a/1b knockout (KO) mice at steady state. **A** Plasma time-concentration profiles of eletriptan in wild type mice (WT (ELE)), mdr1a/1b knockout mice (Mdr1a/1b KO (ELE)), or diphenhydramine in wild type mice (WT (DIPH)) during a 2-h constant intravenous infusion. **B** Total concentrations of eletriptan and diphenhydramine in plasma or brain at steady state. **C** Total brain-to-plasma partitioning coefficient (K_p_). **D** Unbound brain-to-plasma partitioning coefficient (K_p,uu_). The dotted line represents the K_p,uu_ of unity. All data points or columns represent *mean* ± *SD (n* = *3)*. Data were analysed using a one-way ANOVA followed by a Tukey’s multiple comparison test. *: P ≤ 0.05. ***: P ≤ 0.001. ****: P ≤ 0.0001

After having established that eletriptan showed both uptake properties in hCMEC/D3 cells consistent with eletriptan being a H^+^/OC antiporter substrate, and P-gp mediated efflux in IPEC-J2 MDR1 cells, we proceeded to investigate the brain uptake of eletriptan in vivo. The total and unbound brain-to-plasma partitioning coefficients (K_p_ and K_p,uu_) were determined in wild type and mdr1a/1b knockout mice at steady state using the combinatory mapping approach [48]. We included the H^+^/OC antiporter substrate diphenhydramine, as a positive control for active uptake via the H^+^/OC antiporter [31]. Steady state conditions were achieved after a bolus dose followed by a 2-h constant infusion giving plasma concentrations of 259 ± 30 ng/mL. The rationale for selecting this dosing regimen was to obtain a constant plasma concentration that is relatively compatible with clinically relevant human peak plasma concentrations of 94–200 ng/mL [3]. Steady state conditions were achieved after a 2-h constant infusion rate (Fig. 6A). We observed low total brain concentrations of eletriptan in wild type mice, but the concentration rose drastically in mdr1a/1b knockout mice supporting the role of P-gp keeping eletriptan out of the brain (Fig. 6B). K_p_ and K_p,uu_ for eletriptan and diphenhydramine are shown in (Fig. 6C and D). In wild type mice, we observed a K_p_ of 0.17 and a K_p,uu_ of 0.04 for eletriptan, which was increased in a statistically significant manner in mdr1a/1b knockout mice to a K_p_ and K_p,uu_ of 6.35 and 1.32 (> 30-fold increase), respectively. The positive control for active uptake via the H^+^/OC antiporter, diphenhydramine, yielded a K_p_ and a K_p,uu_ of 9.20 and 1.72, respectively. A summary of the determined free fractions (f_u_) and neuropharmacokinetic parameters is shown in Table 5.

**Table 5:** Neuropharmacokinetic parameters of eletriptan and diphenhydramine in wild type and mdr1a/1b knockout mice

		Eletriptan		Diphenhydramine
Parameters	Unit	Wild type	mdr1a/1b knockout	Wild type
f_u,plasma_	%	40	40	40
f_u,brain_	%	8.3	8.3	7.5
C_,plasma_	ng/mL	240 ± 18	229 ± 6	288 ± 14
C_,brain_	ng/mL	41 ± 5	1448 ± 175	2640 ± 265
K_p_ (C_,brain_/C_,plasma_)	unitless	0.17 ± 0.016	6.35 ± 0.91	9.20 ± 1.35
K_p,uu_ (C_,brain·_f_u,brain_)/C_,plasma·_f_u,plasma_)	unitless	0.04 ± 0.004	1.32 ± 0.19	1.72 ± 0.25

Data are represented as *mean* ± *SD* (*n* = 3)

## Discussion

The novelty of the present study is that the triptan family of compounds possesses affinity for the H^+^/OC antiporter, a BBB transport mechanism. At least eletriptan was shown to be a novel substrate of this transporter. In vivo, eletriptan accumulated in the brain when the P-gp function was absent, also indicating active uptake via the H^+^/OC antiporter.

### Triptans are a novel group of compounds shown to interact with the H^+^/OC antiporter

The scope of the study was to investigate possible mechanisms for triptan transport across the BBB and evaluate whether the H^+^/OC antiporter and P-gp contributed to brain uptake and efflux, respectively. Except for frovatriptan, all triptans demonstrated an inhibitory effect on the H^+^/OC antiporter with IC_50_ values ranging from 15 to 1729 µM. Eletriptan displayed the greatest inhibitory potency on pyrilamine uptake among the different triptans, thus indicating the strongest H^+^/OC antiporter interaction. Additionally, eletriptan uptake could almost be completely inhibited in the presence of the prototypical substrate, pyrilamine. The potent inhibitory potential of eletriptan on pyrilamine uptake and vice versa, further point towards that these two compounds represent ideal competitive model substrates for future investigations of the H^+^/OC antiporter. The demonstrated uptake properties of the three investigated triptans, eletriptan, almotriptan, and sumatriptan are summarized in Table 6.

**Table 6:** Overview of the demonstrated uptake properties of eletriptan, almotriptan and sumatriptan into hCMEC/D3 cells

	Eletriptan	Almotriptan	Sumatriptan
IC_50_ value on pyrilamine uptake	IC_50_ = 15 ± 4 µM	IC_50_ = 305 ± 83 µM	IC_50_ = 1458 ± 189 µM
K_m_ value	K_m_ = 89 ± 38 µM	not saturable (in tested range)	not saturable (in tested range)
Inhibition by oxycodone ^#^	yes (*)	no	yes (***)
Inhibition by pyrilamine ^#^	yes (****)	yes (*)	yes (**)
Proton-gradient dependence	yes	yes	yes
Association with H^+^/OC antiporter	Substrate potent inhibitor	(substrate) decent inhibitor	(substrate) very poor inhibitor

^#^ Uptake of the triptans in the absence or presence of oxycodone or pyrilamine were compared using a one-way ANOVA followed by a Tukey’s multiple comparison test

*:P ≤ 0.05. **: P ≤ 0.01. ***: P ≤ 0.001. ****: P ≤ 0.0001

Eletriptan displayed saturable uptake kinetics into hCMEC/D3 cells, while the cellular uptake of almotriptan and sumatriptan revealed no saturation at the tested concentration range. Eletriptan, almotriptan, and sumatriptan uptake were driven by a proton-dependent mechanism, which could indicate the involvement of the H^+^/OC antiporter. In addition to changing the proton driving force of a potential transporter, pH manipulation also affects the protonation degree of the compounds (pK_a_ 8.4–9.6, refer Table 1). Changing the protonation degree of the triptans may affect their passive diffusion properties as well as their affinity towards the transporter, since a protonated amine moiety has shown to be essential for the H^+^/OC antiporter recognition [20, 33]. Thus, the data from the extracellular pH manipulation experiments must be interpreted with caution (Fig. 4). However, as intracellular pH manipulation also showed a tendency towards a pH-dependent uptake mechanism, we expect changes to the observed uptake of eletriptan, almotriptan, and sumatriptan to be a result of the proton gradient and not solely due to changes in the protonation degree of the triptans.

At least eletriptan was found to be a novel substrate for the H^+^/OC antiporter in the present study, while almotriptan, sumatriptan, rizatriptan, naratriptan, and zolmitriptan showed inhibitory affinity towards the transporter.

In agreement with our findings, Doetsch et al. [49] recently proposed that eletriptan might be a substrate of the H^+^/OC antiporter based on in vitro trans-stimulation screening in hCMEC/D3 cells using diphenhydramine as a H^+^/OC antiporter substrate [49]. Frovatriptan and zolmitriptan were also included in that study but did not appear to be H^+^/OC antiporter substrate candidates [49]. Doetsch et al. further evaluated whether eletriptan, frovatriptan, and zolmitriptan were substrates of the organic cation transporter 1 (OCT1). Their findings suggested that frovatriptan and zolmitriptan were substrates of OCT1, while eletriptan was not [49]. In addition, Cheng et al. (2012) have suggested the organic anion-transporting polypeptide 1A2 (OATP1A2) as a carrier with the potential to transport triptans across the BBB [50].

### Eletriptan undergoes simultaneous active uptake and active efflux across the BBB

In the present study, we only identified eletriptan as a P-gp substrate among the triptan family. However, other members of the triptan family have previously been identified as P-gp substrates [51–53]. In the present study, we demonstrated an ER of 28.9 ± 1.8 for eletriptan in IPEC-J2 MDR1 cells. Mahar et al. [53] have previously identified eletriptan (ER of 44.7) and zolmitriptan (ER of 2.48) to be P-gp substrates in the MDR1-transfected Madin Darby canine kidney type II (MDR1-MDCKII) cell line, whereas sumatriptan was not [53]. Additionally, Wilt et al. [51] demonstrated P-gp-mediated efflux of eletriptan and sumatriptan, most pronounced for eletriptan, using indirect measurement of ATP hydrolysis in P-gp-integrated liposomes from wild type mice [51]. Evans et al. [52] assessed the in vivo brain K_p_ in wild type and mdr1a knockout mice for sumatriptan, zolmitriptan, naratriptan, rizatriptan, and eletriptan [52]. Their findings revealed that rizatriptan, naratriptan, and eletriptan were indeed substrates of P-gp, while sumatriptan and zolmitriptan were not.

As demonstrated in the present study, eletriptan underwent active uptake and active efflux in hCMEC/D3 and IPEC-J2 MDR1 cells, respectively. We evaluated this two-way transport phenomenon mediated by two distinct transporters in vivo in both wild type and mdr1a/1b knockout mice. We demonstrated low brain uptake of eletriptan in wild type mice, but with a drastically higher uptake in mrd1a/1b knockout mice (> 30-fold increase), which clearly imply P-gp involvement. Eletriptan demonstrated a K_p,uu_ higher than unity (1.32) in mdr1a/1b knockouts indicating an active uptake mechanism at the BBB. The active uptake component was completely abolished by P-gp in wild type mice indicating poor permeation properties or dominating efflux across the BBB.

The fact that the free brain concentration of eletriptan increased to values higher than the free plasma concentration in the mdr1a/1b knockout mice indicated that (1) eletriptan have a high baseline permeability across the BBB in the absence of the P-gp function, and (2) P-gp is instrumental in keeping brain eletriptan concentrations low in wild type mice. The eletriptan K_p,uu_ values above unity also supported that the high baseline permeability is due to an energy-dependent transporter since an uphill concentration gradient is maintained. This was confirmed by comparing eletriptan K_p,uu_ with the control substrate diphenhydramine.

Diphenhydramine was included as a positive control for active uptake via the H^+^/OC antiporter. Previous microdialysis studies in rats, have reported K_p,uu_ values between 3.24–5.5 for diphenhydramine, which clearly indicate active uptake across the BBB [30–32]. In our study design, where we estimate K_p,uu_ in experiments on mice, using the K_p_ value and calculated free concentrations, diphenhydramine displayed a K_p,uu_ of 1.72. Although the values differ somewhat, they are indicative of H^+^/OC antiporter-mediated active uptake. The differences may be due to species, or a methodological bias. Eletriptan did also display accumulation (K_p,uu_ of 1.32) in the parenchyma in the knock-out mice where the P-gp function was absent. Our in vitro and in vivo findings support the notion that eletriptan is subjected to simultaneous influx and efflux, likely facilitated by the putative H^+^/OC antiporter and P-gp.

### Clinical implications

Inhibition of pyrilamine uptake differed substantially among the triptans, not reflecting the apparent homogeneity in physicochemical properties (Table 1 and Additional file 7). However, triptans also exhibit marked differences in their clinical efficacy and tolerability profiles within and among patients [8, 54]. We show that the affinity for both the H^+^/OC antiporter and the P-gp efflux pump seems to differ considerably among the triptans **(**Figs. 2 and 5A**)**. This observation could potentially provide some explanation on the documented differences in clinical efficacy and tolerability. Furthermore, our findings emphasize the potential of transporter-related drug-drug interactions, when co-administering triptans with substrates of either the H^+^/OC antiporter or P-gp. However, the apparent K_m_ of eletriptan found in hCMEC/D3 cells is much higher than clinically relevant plasma concentrations (246–523 nM) [3]. This may indicate that eletriptan is not saturating the H^+^/OC antiporter in vivo and is therefore not likely to cause transporter associated drug-drug interactions.

Further studies are essential to determine to what extent triptans enter the brain at clinically relevant concentrations and elucidate triptans potential of initiating a central 5HT_1B/1D_ receptor-mediated response. A previous study conducted by Deen et al. [11] demonstrated that the administration of sumatriptan in humans resulted in a notable reduction in central 5HT_1B_ receptor binding. This outcome suggests that sumatriptan exert a pharmacological effect in the brain [11]. Whether central 5HT_1B/1D_ receptor activation leads to anti-migraine and/or potential side effects are still to be elucidated.

## Conclusions

The present study explored the involvement of the putative H^+^/OC antiporter and the P-gp efflux pump in BBB transport of seven clinically relevant triptans. To our knowledge, we are the first to demonstrate that the triptan family of compounds possesses affinity for the H^+^/OC antiporter. We propose that the putative H^+^/OC antiporter might play a role in the BBB transport of some triptans, particularly eletriptan. We confirmed previous studies showing that eletriptan is a substrate of P-gp. Neuropharmacokinetic assessment of eletriptan demonstrated low brain uptake in wild type mice but with a drastically increased uptake in mdr1a/1b knockouts. These findings suggest that eletriptan is subject to simultaneous brain uptake and efflux, possibly facilitated by the putative H^+^/OC antiporter and P-gp, respectively.

In conclusion, this study sheds light on the involvement of the H^+^/OC antiporter and P-gp for brain parenchymal uptake of triptans at the BBB level. These findings may contribute to a better understanding of the ability of triptans to bear a central site of action as well as emphasizing potential transporter-related drug-drug interactions.

### Supplementary Information

**Additional file 1: **Time-dependent uptake of eletriptan and almotriptan into hCMEC/D3 cells. **A **Time-dependent uptake of eletriptan (50 µM) in the absence (Control) or presence of 1000 µM pyrilamine (+pyrilamine) $mean\pm SEM$ (*n* = 4*, N*_*total*_ = 12). **B **Time-dependent uptake of almotriptan (500 µM) in the absence (Control) or presence of 1000 µM pyrilamine (+pyrilamine). Uptake amounts are normalized to protein amount per well. Each data point represents *mean ± SD *(*n* = 3*, N*_*total*_ = 9).

**Additional file 2: **HPLC parameters for almotriptan, eletriptan, sumatriptan, and oxycodone.

**Additional file 3: **MS/MS conditions for almotriptan, eletriptan, sumatriptan, and oxycodone

**Additional file 4: **Cellular uptake of the prototypical H^+^/OC antiporter substrates, [^3^H]-pyrilamine and oxycodone, after intra- and extracellular pH manipulation in hCMEC/D3 cells. **A** Effect of intracellular pH manipulation on [^3^H]-pyrilamine uptake. Uptake of [^3^H]-pyrilamine (1 µCi/mL, 48 nM) was investigated in transport buffer pH 7.4 (Control), after acute exposure to NH_4_Cl (NH_4_Cl (acute)), and after preexposure to NH_4_Cl followed by media change (NH_4_Cl (pre)). **B** Effect of intracellular pH manipulation on oxycodone uptake. Uptake of oxycodone (500 µM) was investigated in transport buffer pH 7.4 (Control), after acute exposure to NH_4_Cl (NH_4_Cl (acute)), and after preexposure to NH_4_Cl followed by media change (NH_4_Cl (pre)). **C** Effect on extracellular pH manipulation on [^3^H]-pyrilamine uptake. Uptake of [^3^H]-pyrilamine (1µCi/mL, 48 nM) was investigated in transport buffer with pH 7.4 (Control), pH 6.8 and pH 8.2 **D** Effect on extracellular pH manipulation on oxycodone uptake. Uptake of oxycodone (500 µM) was investigated in transport buffer with pH 7.4 (Control), pH 6.8 and pH 8.2. Each column represents *mean ± SD* (*n* = 3*, N*_*total*_ = 9). Data were analyzed using a one-way ANOVA followed by a Tukey’s multiple comparison test. **: P ≤ 0.01. ***: P ≤ 0.001. ****: P ≤ 0.0001.

**Additional file 5: **Intracellular pH measurements using BCECF-AM probe. **A **Schematic illustration of BCECF-AM as pH indicator. The cell permeable BCECF-AM enters the cells, where BCECF-AM will undergo hydrolysis to the impermeable BCECF. Fluorescence signal at excitation wavelength of 480 and an emission wavelength of 520 (Ex480/Em520) depends on intracellular pH. An increase in intracellular pH will increase the fluorescent signal, whereas a decrease in intracellular pH will reduce the fluorescent signal. **B **Fluorescent microscopy of BCECF-AM loaded hCMEC/D3 cells. The BCECF-AM dye was loaded in concentrations of 0, 0.5, 1, and 5 µM. Scale bar represents 100 µm. (*n* = 1) **C-D** Changes in intracellular pH were measured over time in BCECF-AM- loaded hCMEC/D3 cells (5 µM) after **C** acute administration of 30 mM NH_4_Cl. 30 mM NH_4_Cl was added automatically after 15 seconds baseline measurements or **D **preincubation with NH_4_Cl in 30-60 minutes followed by manual buffer change to hHBSS(-) without NH_4_Cl. No buffer change control represents the baseline. (*n* = 3*, N*_*total*_ = 9). Each data point represents *mean ± SD.*

**Additional file 6: **Flux curves of eletriptan across IPEC-J2 MDR1 monolayers. The accumulated amounts of eletriptan (50 µM) in the absence or presence of ZSQ (2 µM) in the receiver chamber from apical to basolateral compartment (A–B) or basolateral to apical compartment (B–A). Each data point represents *mean ± SD *(*n* = 3*, N*_*total*_ = 9).

**Additional file 7: **Screening of triptans inhibitory effect on [^3^H]-pyrilamine uptake into hCMEC/D3 cells. **A** Uptake of [^3^H]-pyrilamine (48 nM) in the absence (Control) or presence of 500 µM oxycodone (OXY), eletriptan (ELE), almotriptan (ALMO), rizatriptan (RIZA), zolmitriptan (ZOLMI), naratriptan (NARA), sumatriptan (SUMA) or frovatriptan (FROVA). Each column represents $mean\pm SD$ (*n* = 3*, N*_*total*_ = 9). Columns were compared to ’Control-group’ and analysed using a one-way ANOVA followed by a Tukey’s multiple comparison test. *: P ≤ 0.05. ***: P ≤ 0.001. ****: P ≤ 0.0001.

## Data Availability

All data generated or analyzed during this study are included in this published article and its supplementary information files.

## Abbreviations

5HT_1B__/1D_ receptor: 5-Hydroxytryptamine receptor 1B/1D
A-B: Apical-to-basolateral
B-A: Basolateral-to-apical
BBB: Blood–brain barrier
BCECF-AM: 2′,7′- Bis-(Carboxyethyl)-5(6′)-carboxyfluorescein acetoxymethyl ester
BSA: Bovine serum albumin
CNS: Central nervous system
ER: Efflux ratio
FBS: Fetal bovine serum
f_u,plasma_: Free fraction in plasma
f_u,brain_: Free fraction in brain
HBSS: Hank’s balanced salt solution
hCMEC/D3: Human cerebral microvascular endothelial cell line D3
HEPES: N-2-hydroxyethylpiperazine-N-2-ethane sulfonic acid
H^+^/OC antiporter: Proton-coupled organic cation antiporter
IC_50_: Inhibitory constant at 50% inhibition
IPEC-J2: Intestinal porcine epithelial cell line J2
J_max_: Maximal uptake flux
K_m_: Michaelis Menten constant
KO: Knockout
K_p_: Total brain-to-total plasma concentration ratio
K_p,uu_: Unbound brain-to-unbound plasma concentration ratio
MDCKII: Madin Darby canine kidney type II
MDR1: Human gene encoding P-gp
mdr1a/1b: Rodent gene encoding P-gp
NH_4_Cl: Ammonium chloride
OATP1A2: Organic anion-transporting polypeptide 1A2
OCT1: Organic cation transporter 1
Papp: Apparent permeability
P-gp: P-glycoprotein
SD: Standard deviation
SEM: Standard error of mean
TEER: Transepithelial electrical resistance
WT: Wild type
ZSQ: Zosuquidar

## References

[1] Ashina M, Katsarava Z, Do TP, Buse DC, Pozo-Rosich P, Özge A (2021). Migraine: epidemiology and systems of care. Lancet.

[2] Amiri P, Kazeminasab S, Nejadghaderi SA, Mohammadinasab R, Pourfathi H, Araj-Khodaei M (2021). Migraine: a review on its history, global epidemiology, risk factors, and comorbidities. Front Neurol.

[3] de Vries T, Villalon CM, MaassenVanDenBrink A (2020). Pharmacological treatment of migraine: CGRP and 5-HT beyond the triptans. Pharmacol Ther.

[4] Benemei S, Cortese F, Labastida-Ramirez A, Marchese F, Pellesi L, Romoli M (2017). Triptans and CGRP blockade—impact on the cranial vasculature. J Headache Pain.

[5] de Vries T, Villalón CM, MaassenVanDenBrink A (2020). Pharmacological treatment of migraine: CGRP and 5-HT beyond the triptans. Pharmacol Ther.

[6] Cottier KE, Vanderah TW, Largent-Milnes TM (2017). The CNS as a primary target for migraine therapeutics. Curr Topics Pharmacol.

[7] Tfelt-Hansen PC (2010). Does sumatriptan cross the blood-brain barrier in animals and man?. J Headache Pain.

[8] Dodick DW, Martin V (2004). Triptans and CNS side-effects: pharmacokinetic and metabolic mechanisms. Cephalalgia.

[9] Ferrari MD, Goadsby PJ, Roon KI, Lipton RB (2002). Triptans (serotonin, 5-HT1B/1D agonists) in migraine: detailed results and methods of a meta-analysis of 53 trials. Cephalalgia.

[10] Muzzi M, Zecchi R, Ranieri G, Urru M, Tofani L, De Cesaris F (2019). Ultra-rapid brain uptake of subcutaneous sumatriptan in the rat: Implication for cluster headache treatment. Cephalalgia.

[11] Deen M, Hougaard A, Hansen HD, Schain M, Dyssegaard A, Knudsen GM (2019). Association between Sumatriptan treatment during a migraine attack and central 5-HT1B Receptor Binding. JAMA Neurol.

[12] Varnäs K, Jučaite A, McCarthy DJ, Stenkrona P, Nord M, Halldin C (2013). A PET study with [11C]AZ10419369 to determine brain 5-HT1B receptor occupancy of Zolmitriptan in healthy male volunteers. Cephalalgia.

[13] Sakai Y, Dobson C, Diksic M, Aubé M, Hamel E (2008). Sumatriptan normalizes the migraine attack-related increase in brain serotonin synthesis. Neurology.

[14] Wall A, Kågedal M, Bergström M, Jacobsson E, Nilsson D, Antoni G (2005). Distribution of Zolmitriptan into the CNS in healthy volunteers. Drugs R D.

[15] Dobson CF, Tohyama Y, Diksic M, Hamel E (2004). Effects of acute or chronic administration of anti-migraine drugs Sumatriptan and Zolmitriptan on serotonin synthesis in the rat brain. Cephalalgia.

[16] Johnson DE, Rollema H, Schmidt AW, McHarg AD (2001). Serotonergic effects and extracellular brain levels of eletriptan, zolmitriptan and sumatriptan in rat brain. Eur J Pharmacol.

[17] Goldberg MJ, Spector R, Chiang CK (1987). Transport of diphenhydramine in the central nervous system. J Pharmacol Exp Ther.

[18] Yamazaki M, Fukuoka H, Nagata O, Kato H, Ito Y, Terasaki T (1994). Transport mechanism of an H1-antagonist at the blood-brain barrier: transport mechanism of mepyramine using the carotid injection technique. Biol Pharm Bull.

[19] Okura T, Hattori A, Takano Y, Sato T, Hammarlund-Udenaes M, Terasaki T (2008). Involvement of the pyrilamine transporter, a putative organic cation transporter, in blood-brain barrier transport of oxycodone. Drug Metab Dispos.

[20] Sachkova A, Jensen O, Dücker C, Ansari S, Brockmöller J (2022). The mystery of the human proton-organic cation antiporter: one transport protein or many?. Pharmacol Ther.

[21] Shimomura K, Okura T, Kato S, Couraud PO, Schermann JM, Terasaki T (2013). Functional expression of a proton-coupled organic cation (H+/OC) antiporter in human brain capillary endothelial cell line hCMEC/D3, a human blood-brain barrier model. Fluids Barriers CNS.

[22] Kurosawa T, Tega Y, Uchida Y, Higuchi K, Tabata H, Sumiyoshi T (2022). Proteomics-based transporter identification by the PICK method: involvement of TM7SF3 and LHFPL6 in proton-coupled organic cation antiport at the blood-brain barrier. Pharmaceutics.

[23] Kubo Y, Kusagawa Y, Tachikawa M, Akanuma S, Hosoya K (2013). Involvement of a novel organic cation transporter in verapamil transport across the inner blood-retinal barrier. Pharm Res.

[24] Kurosawa T, Tega Y, Higuchi K, Yamaguchi T, Nakakura T, Mochizuki T (2018). Expression and functional characterization of drug transporters in brain microvascular endothelial cells derived from human induced pluripotent stem cells. Mol Pharmaceut.

[25] Kitamura A, Higuchi K, Okura T, Deguchi Y (2014). Transport characteristics of tramadol in the blood-brain barrier. J Pharm Sci.

[26] Higuchi K, Kitamura A, Okura T, Deguchi Y (2015). Memantine transport by a proton-coupled organic cation antiporter in hCMEC/D3 cells, an in vitro human blood-brain barrier model. Drug Metab Pharmacokinet.

[27] Okura T, Ito R, Ishiguro N, Tamai I, Deguchi Y (2007). Blood-brain barrier transport of pramipexole, a dopamine D2 agonist. Life Sci.

[28] Tega Y, Akanuma S, Kubo Y, Terasaki T, Hosoya K (2013). Blood-to-brain influx transport of nicotine at the rat blood-brain barrier: involvement of a pyrilamine-sensitive organic cation transport process. Neurochem Int.

[29] Kurosawa T, Higuchi K, Okura T, Kobayashi K, Kusuhara H, Deguchi Y (2017). Involvement of proton-coupled organic cation antiporter in varenicline transport at blood-brain barrier of rats and in human brain capillary endothelial cells. J Pharm Sci.

[30] Kawase A, Chuma T, Irie K, Kazaoka A, Kakuno A, Matsuda N (2021). Increased penetration of diphenhydramine in brain via proton-coupled organic cation antiporter in rats with lipopolysaccharide-induced inflammation. Brain Behav Immunity Health.

[31] Sadiq MW, Borgs A, Okura T, Shimomura K, Kato S, Deguchi Y (2011). Diphenhydramine active uptake at the blood-brain barrier and its interaction with oxycodone in vitro and in vivo. J Pharm Sci.

[32] Kitamura A, Okura T, Higuchi K, Deguchi Y (2016). Cocktail-dosing microdialysis study to simultaneously assess delivery of multiple organic-cationic drugs to the brain. J Pharm Sci.

[33] Smirnova MA, Goracci L, Cruciani G, Federici L, Declèves X, Chapy H (2022). Pharmacophore-based discovery of substrates of a novel drug/proton-antiporter in the human brain endothelial hCMEC/D3 cell line. Pharmaceutics.

[34] Chapy H, Goracci L, Vayer P, Parmentier Y, Carrupt P-A, Declèves X (2015). Pharmacophore-based discovery of inhibitors of a novel drug/proton antiporter in human brain endothelial hCMEC/D3 cell line. Brit J Pharmacol.

[35] Chapy H, Smirnova M, Andre P, Schlatter J, Chiadmi F, Couraud PO (2014). Carrier-mediated cocaine transport at the blood-brain barrier as a putative mechanism in addiction liability. Int J Neuropsychopharmacol.

[36] Weksler B, Romero IA, Couraud P-O (2013). The hCMEC/D3 cell line as a model of the human blood brain barrier. Fluids Barriers CNS.

[37] Saaby L, Helms HC, Brodin B (2016). IPEC-J2 MDR1, a novel high-resistance cell line with functional expression of human P-glycoprotein (ABCB1) for Drug Screening Studies. Mol Pharm.

[38] Boron WF, De Weer P (1976). Intracellular pH transients in squid giant axons caused by CO2, NH3, and metabolic inhibitors. J Gen Physiol.

[39] Eneberg E, Jones CR, Jensen T, Langthaler K, Bundgaard C (2022). Practical application of rodent transporter knockout models to assess brain penetration in drug discovery. Drug Metab Bioanal Lett.

[40] Langthaler K, Jones CR, Brodin B, Bundgaard C (2023). Assessing extent of brain penetration in vivo (Kp, uu, brain) in Göttingen minipig using a diverse set of reference drugs. Eur J Pharm Sci.

[41] Rubio-Beltrán E, Labastida-Ramírez A, Villalón CM, MaassenVanDenBrink A (2018). Is selective 5-HT1F receptor agonism an entity apart from that of the triptans in antimigraine therapy?. Pharmacol Ther.

[42] DrugBank online. Almotriptan DB00918; Eletriptan DB00216, Frovatriptan DB00998, Naratriptan DB00952, Rizatriptan DB00953, Sumatriptan DB00669, Zolmitriptan DB00315, Oxycodone DB00497, Pyrilamine DB06691. https://go.drugbank.com/drugs. Accessed 23 Feb 2023.

[43] PubChem. PubChem Compound Summary for: Almotriptan; Eletriptan; Frovatriptan; Naratriptan; Sumatriptan; Zolmitriptan; Oxycodone; Pyrilamine. https://pubchem.ncbi.nlm.nih.gov. Accessed 23 Feb 2023.

[44] ChemSpider. Almotriptan; Eletriptan; Frovatriptan; Naratriptan; Rizatriptan; Sumatriptan; Zolmitriptan; Oxycodone; Pyrilamine. https://www.chemspider.com/. Accessed 23 Feb 2023.

[45] Leslie EM, Deeley RG, Cole SP (2005). Multidrug resistance proteins: role of P-glycoprotein, MRP1, MRP2, and BCRP (ABCG2) in tissue defense. Toxicol Appl Pharmacol.

[46] Sharom FJ (2008). ABC multidrug transporters: structure, function and role in chemoresistance. Pharmacogenomics.

[47] Ozgur B, Saaby L, Langthaler K, Brodin B (2018). Characterization of the IPEC-J2 MDR1 (iP-gp) cell line as a tool for identification of P-gp substrates. Eur J Pharm Sci.

[48] Loryan I, Sinha V, Mackie C, Van Peer A, Drinkenburg W, Vermeulen A (2014). Mechanistic understanding of brain drug disposition to optimize the selection of potential neurotherapeutics in drug discovery. Pharm Res.

[49] Doetsch DA, Ansari S, Jensen O, Gebauer L, Dücker C, Brockmöller J (2022). Substrates of the human brain proton-organic cation antiporter and comparison with organic cation transporter 1 activities. Int J Mol Sci.

[50] Cheng Z, Liu H, Yu N, Wang F, An G, Xu Y (2012). Hydrophilic anti-migraine triptans are substrates for OATP1A2, a transporter expressed at human blood-brain barrier. Xenobiotica.

[51] Wilt LA, Nguyen D, Roberts AG (2017). Insights into the molecular mechanism of triptan transport by P-glycoprotein. J Pharm Sci.

[52] Evans DC, O'Connor D, Lake BG, Evers R, Allen C, Hargreaves R (2003). eletriptan metabolism by human hepatic cyp450 enzymes and transport by human P-glycoprotein. Drug Metab Dispos.

[53] Mahar Doan KM, Humphreys JE, Webster LO, Wring SA, Shampine LJ, Serabjit-Singh CJ (2002). Passive permeability and P-glycoprotein-mediated efflux differentiate central nervous system (CNS) and non-CNS marketed drugs. J Pharmacol Exp Ther.

[54] Tfelt-Hansen P, Edvinsson L (2007). Pharmacokinetic and pharmacodynamic variability as possible causes for different drug responses in migraine. Comment Cephalalgia.

