# Supplementary figures and images for: The putative proton-coupled organic cation antiporter is involved in uptake of triptans into human brain capillary endothelial cells
Source: Fluids Barriers CNS. 2024 May 6;21:39. doi: 10.1186/s12987-024-00544-6 (PMC11071266; doi:10.1186/s12987-024-00544-6)

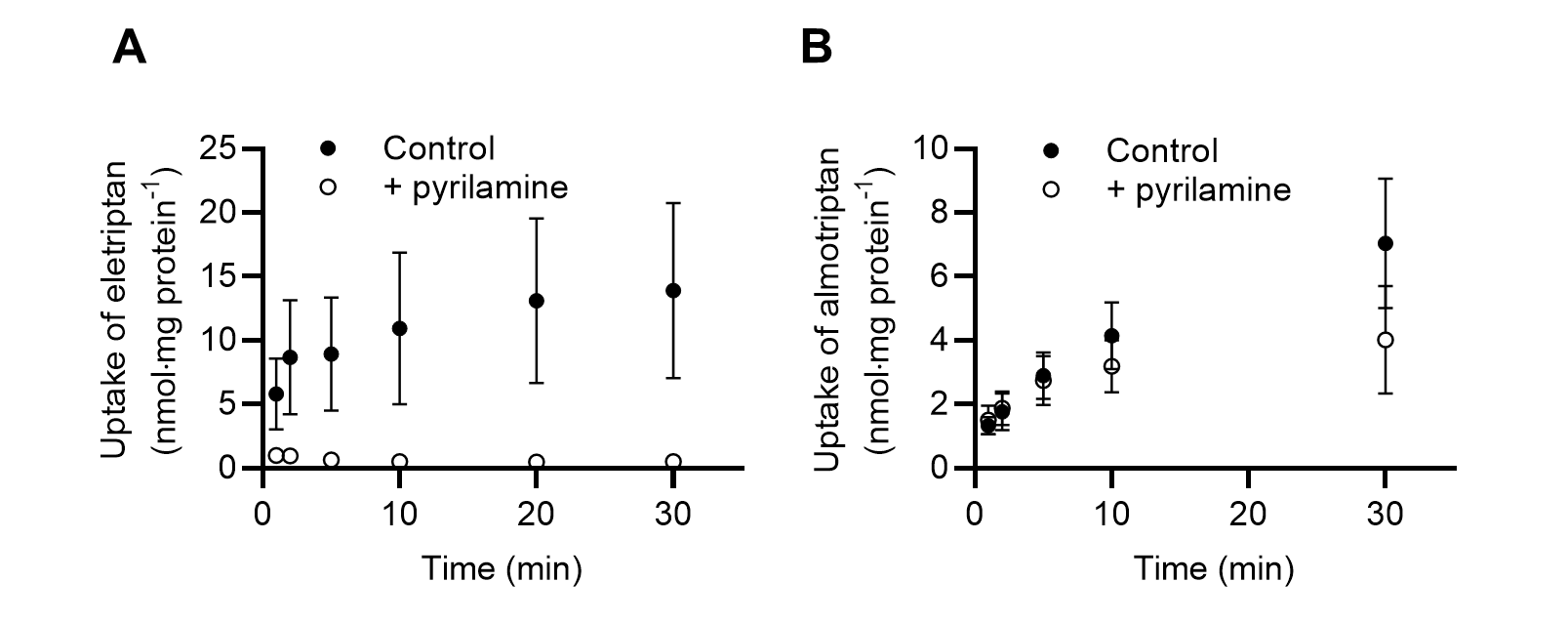

Supplement: Supplementary file 1 — Additional file 1: Time-dependent uptake of eletriptan and almotriptan into hCMEC/D3 cells. A Time-dependent uptake of eletriptan (50 µM) in the absence (Control) or presence of 1000 µM pyrilamine (+pyrilamine) $mean\pm SEM$mean±SEM (n = 4, Ntotal = 12). B Time-dependent uptake of almotriptan (500 µM) in the absence (Control) or presence of 1000 µM pyrilamine (+pyrilamine). Uptake amounts are normalized to protein amount per well. Each data point represents mean ± SD (n = 3, Ntotal = 9). [file 12987_2024_544_MOESM1_ESM.tif]

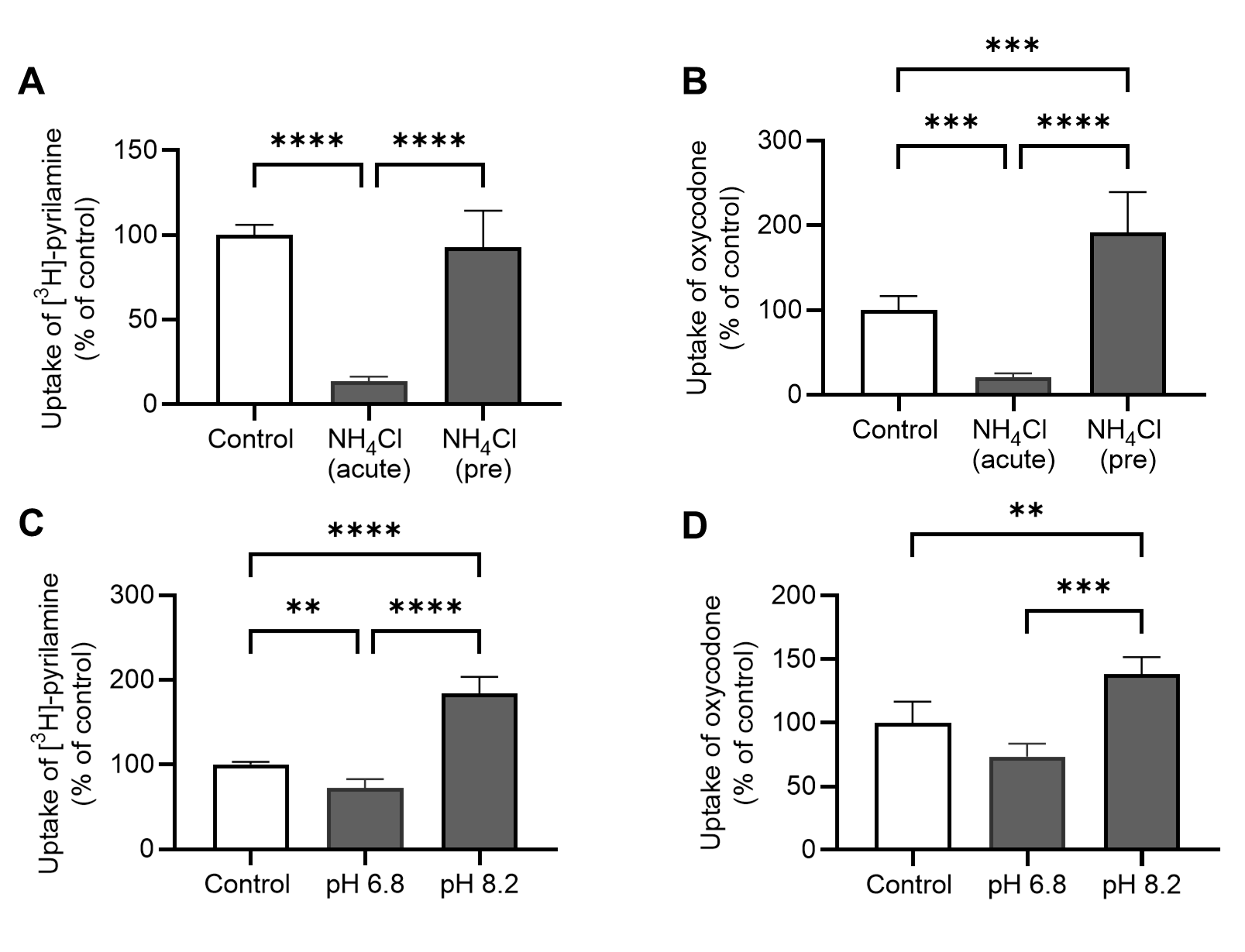

Supplement: Supplementary file 4 — Additional file 4: Cellular uptake of the prototypical H+/OC antiporter substrates, [3H]-pyrilamine and oxycodone, after intra- and extracellular pH manipulation in hCMEC/D3 cells. A Effect of intracellular pH manipulation on [3H]-pyrilamine uptake. Uptake of [3H]-pyrilamine (1 µCi/mL, 48 nM) was investigated in transport buffer pH 7.4 (Control), after acute exposure to NH4Cl (NH4Cl (acute)), and after preexposure to NH4Cl followed by media change (NH4Cl (pre)). B Effect of intracellular pH manipulation on oxycodone uptake. Uptake of oxycodone (500 µM) was investigated in transport buffer pH 7.4 (Control), after acute exposure to NH4Cl (NH4Cl (acute)), and after preexposure to NH4Cl followed by media change (NH4Cl (pre)). C Effect on extracellular pH manipulation on [3H]-pyrilamine uptake. Uptake of [3H]-pyrilamine (1µCi/mL, 48 nM) was investigated in transport buffer with pH 7.4 (Control), pH 6.8 and pH 8.2 D Effect on extracellular pH manipulation on oxycodone uptake. Uptake of oxycodone (500 µM) was investigated in transport buffer with pH 7.4 (Control), pH 6.8 and pH 8.2. Each column represents mean ± SD (n = 3, Ntotal = 9). Data were analyzed using a one-way ANOVA followed by a Tukey’s multiple comparison test. **: P ≤ 0.01. ***: P ≤ 0.001. ****: P ≤ 0.0001. [file 12987_2024_544_MOESM4_ESM.tif]

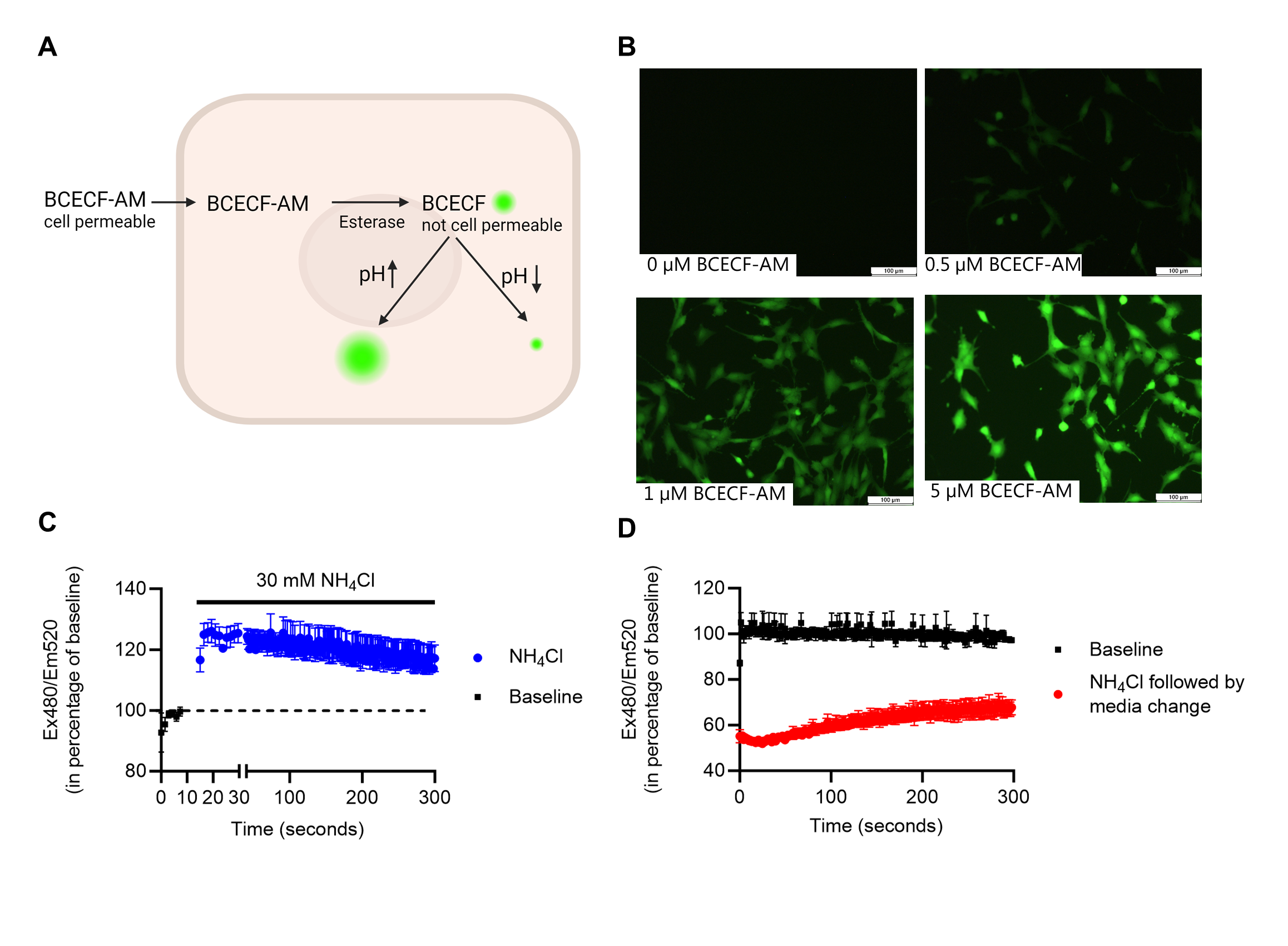

Supplement: Supplementary file 5 — Additional file 5: Intracellular pH measurements using BCECF-AM probe. A Schematic illustration of BCECF-AM as pH indicator. The cell permeable BCECF-AM enters the cells, where BCECF-AM will undergo hydrolysis to the impermeable BCECF. Fluorescence signal at excitation wavelength of 480 and an emission wavelength of 520 (Ex480/Em520) depends on intracellular pH. An increase in intracellular pH will increase the fluorescent signal, whereas a decrease in intracellular pH will reduce the fluorescent signal. B Fluorescent microscopy of BCECF-AM loaded hCMEC/D3 cells. The BCECF-AM dye was loaded in concentrations of 0, 0.5, 1, and 5 µM. Scale bar represents 100 µm. (n = 1) C-D Changes in intracellular pH were measured over time in BCECF-AM- loaded hCMEC/D3 cells (5 µM) after C acute administration of 30 mM NH4Cl. 30 mM NH4Cl was added automatically after 15 seconds baseline measurements or D preincubation with NH4Cl in 30-60 minutes followed by manual buffer change to hHBSS(-) without NH4Cl. No buffer change control represents the baseline. (n = 3, Ntotal = 9). Each data point represents mean ± SD. [file 12987_2024_544_MOESM5_ESM.tif]

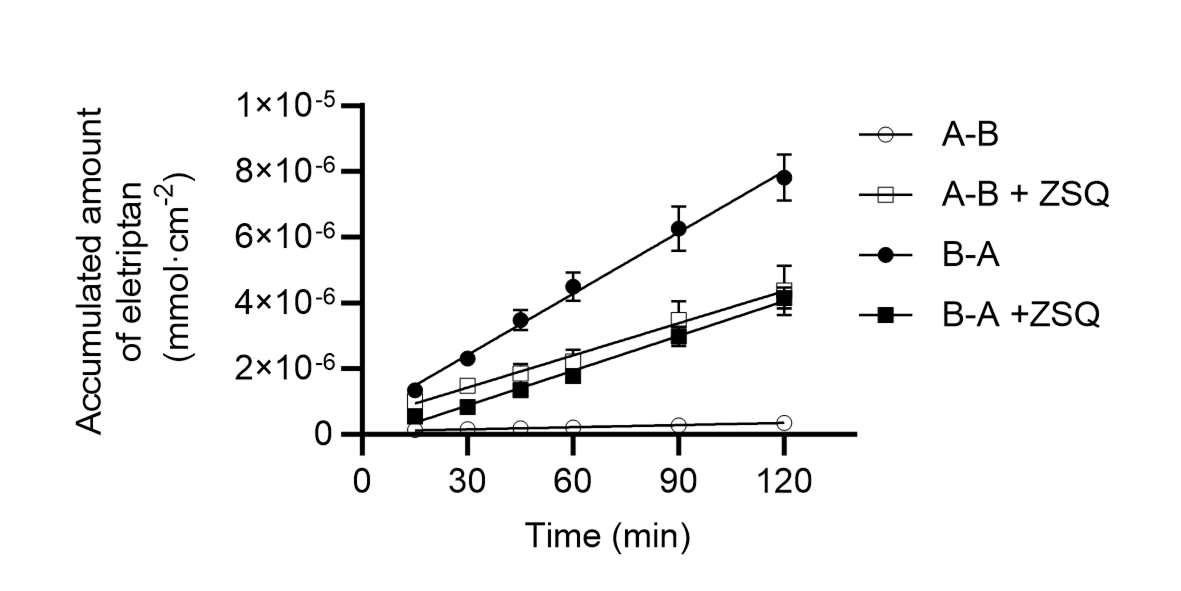

Supplement: Supplementary file 6 — Additional file 6: Flux curves of eletriptan across IPEC-J2 MDR1 monolayers. The accumulated amounts of eletriptan (50 µM) in the absence or presence of ZSQ (2 µM) in the receiver chamber from apical to basolateral compartment (A–B) or basolateral to apical compartment (B–A). Each data point represents mean ± SD (n = 3, Ntotal = 9). [file 12987_2024_544_MOESM6_ESM.tif]

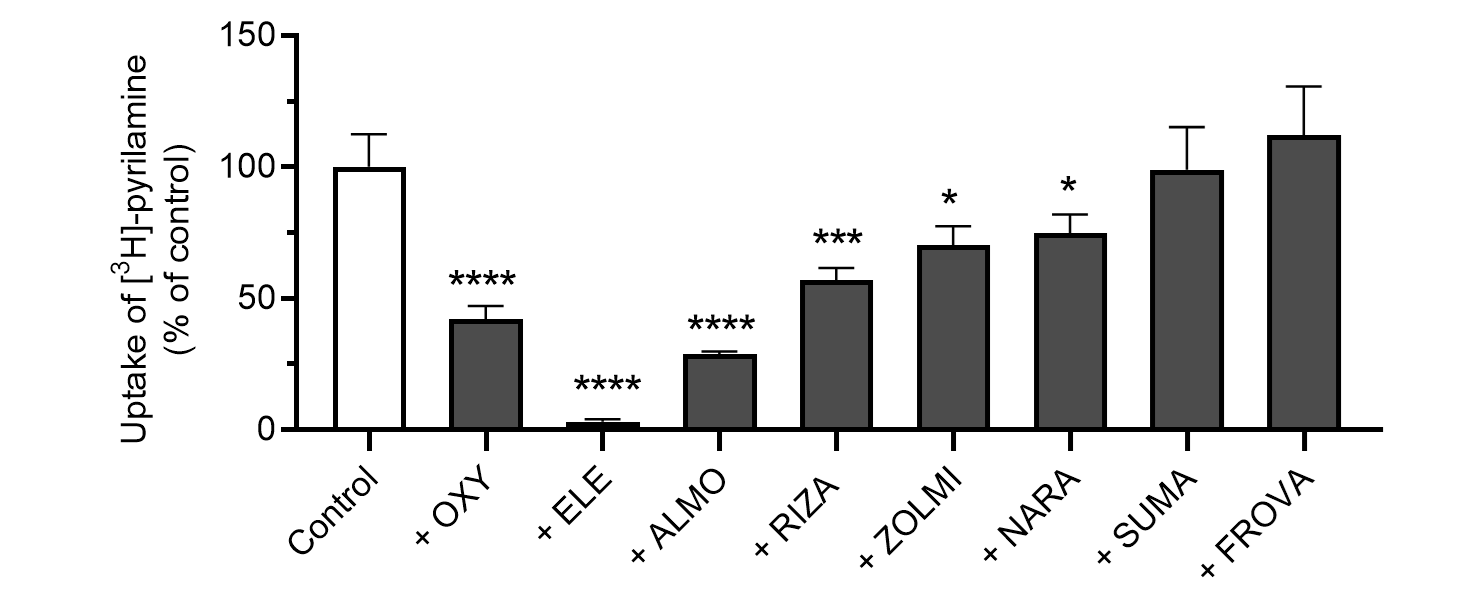

Supplement: Supplementary file 7 — Additional file 7: Screening of triptans inhibitory effect on [3H]-pyrilamine uptake into hCMEC/D3 cells. A Uptake of [3H]-pyrilamine (48 nM) in the absence (Control) or presence of 500 µM oxycodone (OXY), eletriptan (ELE), almotriptan (ALMO), rizatriptan (RIZA), zolmitriptan (ZOLMI), naratriptan (NARA), sumatriptan (SUMA) or frovatriptan (FROVA). Each column represents $mean\pm SD$mean±SD (n = 3, Ntotal = 9). Columns were compared to ’Control-group’ and analysed using a one-way ANOVA followed by a Tukey’s multiple comparison test. *: P ≤ 0.05. ***: P ≤ 0.001. ****: P ≤ 0.0001. [file 12987_2024_544_MOESM7_ESM.tif]
