# Supplementary material for: The putative proton-coupled organic cation antiporter is involved in uptake of triptans into human brain capillary endothelial cells
Source: Fluids Barriers CNS. 2024 May 6;21:39. doi: 10.1186/s12987-024-00544-6 (PMC11071266; doi:10.1186/s12987-024-00544-6)
Supplement: Supplementary file 2 — Additional file 2: HPLC parameters for almotriptan, eletriptan, sumatriptan, and oxycodone. [file 12987_2024_544_MOESM2_ESM.docx]

**Additional file 2. HPLC parameters for almotriptan, eletriptan, sumatriptan, and oxycodone.**

|  | **Almotriptan** | **Eletriptan** | **Sumatriptan** | **Oxycodone** |
| --- | --- | --- | --- | --- |
| Mobile phase | A: 0.1% formic acid in ultrapure.  B: 0.1% formic acid in acetonitrile or methanol. | A: 0.1% formic acid in ultrapure water.  B: 0.1% formic acid in acetonitrile. | A: 0.1% formic acid in ultrapure.  B: 0.1% formic acid in acetonitrile. | A: ultrapure water.  B: acetonitrile. |
| Gradient/ isocratic | 60/40 isocratic elution | 60/40 isocratic elution | 60/40 isocratic elution or gradient | Gradient |
| Flow rate | 0.2 mL/min | 0.3 mL/min | 0.2 or 0.3 mL/min | 0.3 mL/min |
| Injection volume | 5 µL | 5 µL | 5 µL | 10 µL |
