# Supplementary material for: The putative proton-coupled organic cation antiporter is involved in uptake of triptans into human brain capillary endothelial cells
Source: Fluids Barriers CNS. 2024 May 6;21:39. doi: 10.1186/s12987-024-00544-6 (PMC11071266; doi:10.1186/s12987-024-00544-6)
Supplement: Supplementary file 3 — Additional file 3: MS/MS conditions for almotriptan, eletriptan, sumatriptan, and oxycodone [file 12987_2024_544_MOESM3_ESM.docx]

**Additional file 3. MS/MS conditions for almotriptan, eletriptan, sumatriptan, and oxycodone.**

| **Drug** | **Precursor-product ion pair** | | **Cone (V)** | **Collision energy (eV)** | |
| --- | --- | --- | --- | --- | --- |
| Almotriptan | | 336 🡪157.1 | 35 | | 20 |
| Eletriptan | | 383🡪117 | 31 | | 5 |
| Sumatriptan | | 296.15 🡪157.1 | 25 | | 20 |
| Oxycodone | | 315.2 🡪 298.1 | - | | 9 |
